# Listeria monocytogenes TcyKLMN Cystine/Cysteine Transporter Facilitates Glutathione Synthesis and Virulence Gene Expression

**DOI:** 10.1128/mbio.00448-22

**Published:** 2022-04-18

**Authors:** Moran Brenner, Sivan Friedman, Adi Haber, Nurit Livnat-Levanon, Ilya Borovok, Nadejda Sigal, Oded Lewinson, Anat A. Herskovits

**Affiliations:** a The Shmunis School of Biomedicine and Cancer Research, The George S. Wise Faculty of Life Sciences, Tel Aviv University, Tel Aviv, Israel; b Department of Biochemistry, The Bruce and Ruth Rappaport Faculty of Medicine, The Rappaport Institute for Biomedical research, Technion—Israel Institute of Technology, Haifa, Israel; The Hebrew University of Jerusalem

**Keywords:** *Listeria monocytogenes*, glutathione biosynthesis, GSH, cystine/cysteine importer, branched-chain amino acids, BCAA

## Abstract

Listeria monocytogenes is a saprophyte and a human intracellular pathogen. Upon invasion into mammalian cells, it senses multiple metabolic and environmental signals that collectively trigger its transition to the pathogenic state. One of these signals is the tripeptide glutathione, which acts as an allosteric activator of L. monocytogenes’s master virulence regulator, PrfA. While glutathione synthesis by L. monocytogenes was shown to be critical for PrfA activation and virulence gene expression, it remains unclear how this tripeptide is synthesized in changing environments, especially in light of the observation that L. monocytogenes is auxotrophic to one of its precursors, cysteine. Here, we show that the ABC transporter TcyKLMN is a cystine/cysteine importer that supplies cysteine for glutathione synthesis, hence mediating the induction of the virulence genes. Further, we demonstrate that this transporter is negatively regulated by three metabolic regulators, CodY, CymR, and CysK, which sense and respond to changing concentrations of branched-chain amino acids (BCAA) and cysteine. The data indicate that under low concentrations of BCAA, TcyKLMN is upregulated, driving the production of glutathione by supplying cysteine, thereby facilitating PrfA activation. These findings provide molecular insight into the coupling of L. monocytogenes metabolism and virulence, connecting BCAA sensing to cysteine uptake and glutathione biosynthesis as a mechanism that controls virulence gene expression. This study exemplifies how bacterial pathogens sense their intracellular environment and exploit essential metabolites as effectors of virulence.

## INTRODUCTION

Listeria monocytogenes is a Gram-positive, facultative, intracellular pathogen and the causative agent of listeriosis, a disease that can lead to severe clinical manifestations in pregnant women, neonates, and immunocompromised adults (1). L. monocytogenes is characterized by its intracellular lifestyle but can also grow outside the host on soil and vegetation, as well as on food products (2). In the mammalian host, L. monocytogenes invades a wide array of cells (phagocytic and nonphagocytic) by expressing specialized proteins, named internalins, that facilitate its internalization (e.g., InlA and InlB) (3). Upon internalization, the bacteria are initially found within a membrane-bound vacuole, from which they escape into the host cell cytosol via the action of several virulence factors—the pore-forming toxin listeriolysin O (LLO, encoded by the *hly* gene), and the two phospholipases PlcA and PlcB (4–6). In the host cell cytosol, the bacteria utilize host-derived metabolites to support growth (7–10), and using ActA protein, hijack the host actin-polymerization machinery to move around the cell and spread from cell to cell (11, 12). All of the above-mentioned virulence factors as well as many others are positively regulated by PrfA, the master virulence regulator of L. monocytogenes (13, 14).

Multiple metabolic and physiological signals are responsible for the transition of L. monocytogenes from the saprophytic to the pathogenic state (15–20). Many of these signals converge at PrfA and directly or indirectly regulate its transcription, translation, and activity. One of these signals is low concentration of branched-chain amino acids (low BCAA), a condition that is found in the intracellular niche. It was previously demonstrated by our lab that L. monocytogenes responds to low BCAA by upregulating the expression of PrfA, which, in turn, activates the virulence genes (15). This response was shown to depend on the global metabolic regulator CodY, which is also a sensor of BCAA, as it directly binds isoleucine (15, 21, 22). Under high-BCAA conditions, CodY was shown to bind isoleucine and in that form to repress the transcription of many metabolic genes, including those involved in BCAA biosynthesis (21–25). While this was considered the main mechanism of CodY regulation, we demonstrated that in L. monocytogenes CodY is also active under low-BCAA conditions, i.e., in its isoleucine-unbound form, where it up- and downregulates the transcription of many genes, among them *prfA* (upregulating its transcription), hence playing a role in the induction of the virulence genes (15, 22). While these findings demonstrated that PrfA expression is essentially linked to the availability of BCAA in the intracellular niche, they further indicated that sensing of the metabolic host cell cytosol environment is key to the regulation of L. monocytogenes virulence.

Another metabolite that was recently shown to affect L. monocytogenes virulence is glutathione. Glutathione is a low-molecular-weight peptide thiol that is highly abundant in the host cell cytosol in its reduced form (GSH), functioning as a redox buffer, antioxidant, and enzyme cofactor (26). Glutathione is also synthesized by some bacteria, mainly Gram-negative and a few Gram-positive (such as L. monocytogenes), and plays a role in redox homeostasis and bacterial survival under oxidative stress (26). A previous study demonstrated that L. monocytogenes’s glutathione synthetase, GshF, plays a critical role in the induction of the virulence genes during infection (17). GshF is a bifunctional enzyme that catalyzes the two reactions that synthesize the tripeptide glutathione (i.e., l-γ-glutamyl-l-cysteinylglycine) (27). It first ligates the γ-carboxyl group of l-glutamate to l-cysteine, a reaction that is the rate-limiting step of glutathione biosynthesis, and then condenses the product γ-glutamylcysteine with glycine. GSH itself was shown to allosterically bind PrfA and act as its activating cofactor (28, 29). More specifically, the binding of glutathione to PrfA was shown to cause a conformational change that primes its binding to DNA, as shown for other ligand-binding Crp/Fnr transcription regulators (28, 29).

As indicated, cysteine is one of the building blocks of glutathione, and its rate-limiting precursor, however, it is not synthesized by L. monocytogenes. L. monocytogenes lacks the ability to reduce sulfate to sulfide, which is required for cysteine biosynthesis, and hence has to import cysteine from the environment (30). Notably, L. monocytogenes is also auxotrophic to methionine (which also has to be imported), and it cannot synthesize cysteine from methionine, as it lacks the transsulfuration pathway that converts methionine to cysteine (9, 30). To date, two transport systems have been shown to be involved in the acquisition of cysteine by L. monocytogenes. The first is the ABC-transporter Lmo0135-0137 (whose substrate-binding protein is CtaP), which was shown to support the uptake of free cysteine during growth in a synthetic medium (31). The other system is the OppABCDF transporter, which was shown to import oligopeptides, including cysteine-containing peptides, that were shown to serve as a source of cysteine for glutathione synthesis and PrfA activation (20). While these systems were reported to promote to L. monocytogenes invasion and virulence gene expression in mammalian cells, respectively, their contribution to L. monocytogenes intracellular growth was only partial (20, 31, 32), implying that additional systems are involved in the acquisition of cysteine within the intracellular niche.

In this study, we report on TcyKLMN, another ABC-transporter that is directly involved in cysteine uptake in L. monocytogenes. We show that this transporter imports both cystine and cysteine and plays a role in the activation of L. monocytogenes virulence gene expression by supplying cysteine for glutathione synthesis. Further, we demonstrate that this transporter is regulated by CodY, CymR, and CysK, three metabolic factors that act as repressors under nutrient-rich conditions. The findings presented here establish that cysteine import is key to the transition of L. monocytogenes to the pathogenic state and provide another example of the coupling of metabolism and virulence in bacterial pathogens.

## RESULTS

### Genes associated with cysteine uptake and metabolism modulate virulence gene expression.

**FIG 1:**
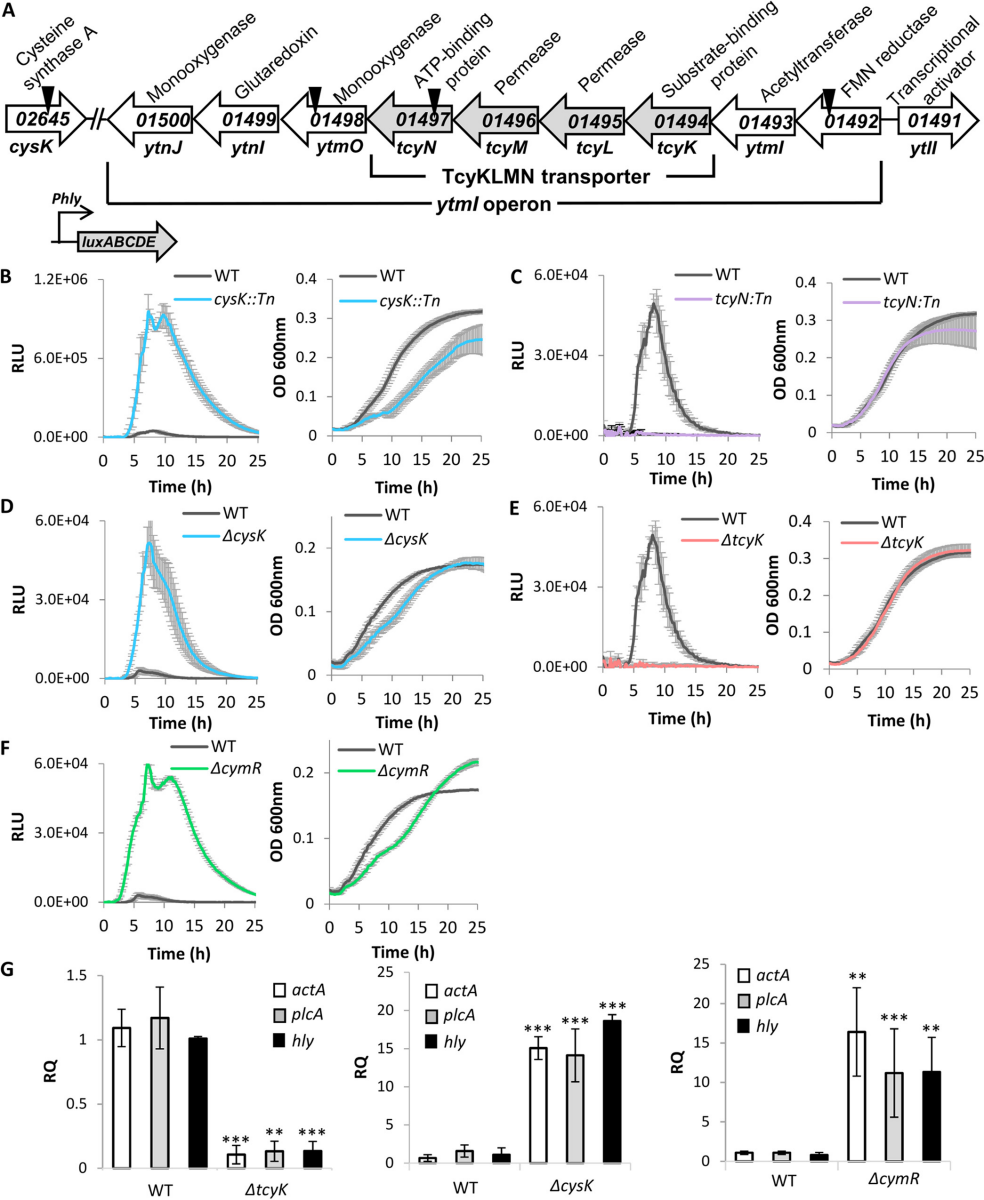
The TcyKLMN transporter and the regulatory factors CysK and CymR play a role in the regulation of L. monocytogenes virulence gene expression. (A) A schematic representation of the *ytmI* operon and the genes identified in Friedman et al. 2017. Locations of transposon insertions are marked with a triangle. (B to F) Luminescence and OD_600_ measurements of bacteria grown in 96-well plates in minimal defined medium supplemented with low concentrations of branched-chain amino acids (LBMM). Relative luminescence units (RLU) represent luminescence values normalized to the respective OD_600_ value. OD_600_ values represent growth. The data represent 3 biological replicates. Error bars indicate standard deviation. (G) qRT-PCR analysis of *actA*, *plcA*, and *hly* transcription levels in indicated bacteria grown in LBMM. mRNA levels were normalized to *rpoD* mRNA and are represented as relative quantity (RQ), relative to the mRNA level in WT bacteria. The data represent 3 biological replicates. Error bars indicate standard deviation. Asterisks represent *P* values (*, *P* < 0.05; **, *P* < 0.01; ***, *P* < 0.001) calculated by Student’s *t* test. *P* values represent a comparison to the BHI sample. The same data of WT L. monocytogenes were used in panels C and E, and D and F, as all of these bacteria were grown on the same 96-well plate. The results were separated for better visualization.

We previously performed a genetic screen of a mariner transposon mutant library in search of genes that differentially regulate the virulence genes of L. monocytogenes strain 10403S under low BCAA. The screen identified multiple genes that were associated with cysteine uptake and metabolism—*tcyN* (*LMRG_01497*), *ytmO* (*LMRG_01498*), *LMRG_01492*, and *cysK* (*LMRG_02645*) (Fig. 1A and Fig. S1A) (33). Interestingly, the first three genes mapped to the *ytmI* operon, which in Bacillus subtilis was shown to encode the cystine ABC-transporter, TcyJKLMN (Fig. 1A; of note, in L. monocytogenes this operon lacks the *tcyJ* gene) (34). *tcyN* encodes the transporter’s ATP-binding protein, whereas *ytmO* and *LMRG_01492* encode a monooxygenase and a flavin mononucleotide (FMN) reductase, respectively, whose functions in cystine uptake/metabolism remain elusive. Apart from this locus, the *cysK* gene was also identified, encoding an *O*-acetylserine thiol-lyase (which is part of the cysteine synthase complex), which plays a role in cysteine regulation and synthesis (Fig. 1A and Fig. S1B). In B. subtilis, CysK was shown to be a trigger enzyme that acts as both a metabolic enzyme and a transcription regulator, the latter via interaction with CymR, a key transcription regulator in cysteine/methionine metabolism (35). It was shown that CymR and CysK form a complex that negatively regulates the *ytmI* operon by repressing the transcription of its activator YtlI (also called AscR) (36, 37), which is encoded upstream to the operon in the opposite direction (a similar gene organization exists in L. monocytogenes; Fig. 1A) (38). Although *ytlI* and *cymR* were not identified in our screen, we found homologues in the L. monocytogenes genome (*LMRG_01491* and *LMRG_01455*, respectively), suggesting that the L. monocytogenes *ytmI* operon is regulated similarly to that of B. subtilis.

10.1128/mbio.00448-22.1 FIG S1 Cysteine uptake and metabolic genes identified in a screen by Friedman et al. (A) Phenotypes of transposon mutants as identified in Friedman et al. WT bacteria and indicated transposon mutants were grown in minimal defined medium supplemented with a low concentration of branched-chain amino acids (LBMM). Relative luminescence units (RLU) represent luminescence values normalized to the respective OD_600_ value. (B) Schematic representation of cysteine transport via TcyKLMN and cysteine-glutathione biosynthesis. The genes identified in the genetic screen are marked in red. OAS, *O*-acetylserine. Download FIG S1, PDF file, 0.7 MB. Copyright © 2022 Brenner et al. 2022 Brenner et al. https://creativecommons.org/licenses/by/4.0/ This content is distributed under the terms of the Creative Commons Attribution 4.0 International license.

To investigate the cysteine-associated genes and their role in the regulation of L. monocytogenes virulence, we first examined whether L. monocytogenes strain 10403S is auxotrophic to cysteine and methionine, as some variations have been reported for different L. monocytogenes strains (30). As shown in Fig. S2, L. monocytogenes strain 10403S is unable to grow in the absence of cysteine or methionine and cannot use either of them as a source for the other, and hence had to be supplied with both. We next confirmed the phenotypes of the mariner transposon mutants, focusing on *cysK*::*Tn* and *tcyN*::*Tn*, using the same assay that was used in the original screen. Virulence gene expression was monitored using a reporter system that expresses the *luxABCDE* genes under the control of the PrfA-regulated *hly* promoter, which was cloned on the integrative plasmid pPL2 (pPL2-*Phly-lux*). Of note, wild-type (WT) bacteria carrying this plasmid grown in a minimal defined medium containing low BCAA (low BCAA minimal medium, LBMM) display an enhanced luminescence profile, which represents the increased transcription of the *hly* gene (previously shown in reference 21). As mentioned, this upregulation of *hly* transcription in LBMM is completely dependent on CodY, as, under this condition, CodY activates the transcription of PrfA (15, 21). Examining the luminescence profiles of *cysK*::*Tn* and *tcyN*::*Tn* during growth in LBMM, we observed that the mutants differentially affect *Phly-lux* expression in comparison to WT bacteria. While the *cysK*::*Tn* mutant exhibited an enhanced luminescence profile, *tcyN*::*Tn* failed to show luminescence signals (Fig. 1 B and C) (33). To validate these phenotypes, we generated clean deletion mutants of *cysK* and *tcyK*, the latter encoding the substrate binding protein (SBP) of TcyKLMN (*LMRG_01494*). SBPs of ABC transporters (specifically importers) are key factors that bind the transported substrate extracellularly and facilitate its import into the cell, hence determining the substrate specificity of the transporter (39). Since both *tcyK* and *tcyN* are essential components of TcyKLMN, we chose to delete *tcyK* instead of *tcyN*, to further investigate the transporter’s substrate specificity. As shown in Fig. 1 D and E, Δ*cysK* and Δ*tcyK* recapitulated the phenotypes of their corresponding transposon mutants. Of note, the mutants grew similarly to WT bacteria in LBMM and in the rich medium brain heart infusion (BHI) (Fig. 1 D and E and Fig. S3A and B). Since in B. subtilis CysK was shown to repress *tcyJKLMN* by forming a complex with CymR, we generated a Δ*cymR* mutant of L. monocytogenes and analyzed its luminescence profile during growth in LBMM using the *Phly-lux* reporter system. The data indicated that Δ*cymR* behaves similarly to Δ*cysK*, i.e., exhibits an enhanced luminescence profile, overall demonstrating that CysK and CymR negatively affect the transcription of *hly* (Fig. 1F and Fig. S3C). To confirm the effects of TcyK, CysK, and CymR on the expression of the virulence genes, the transcription levels of *actA*, *plcA*, and *hly* (three major virulence genes of L. monocytogenes) were evaluated in Δ*tcyK*, Δ*cysK*, and Δ*cymR* mutants and compared to those in WT bacteria grown in LBMM, using reverse transcription-quantitative PCR (qRT-PCR). As shown in Fig. 1G, the transcription level of the virulence genes corroborated the luminescence data, demonstrating a low transcription level in Δ*tcyK* (∼10-fold) and an enhanced transcription level in Δ*cysK* and Δ*cymR* (∼15-fold) in comparison to WT bacteria. These phenotypes were further complemented by introducing a pPL2 plasmid containing a copy of *cysK* or *tcyK* to the corresponding mutants (under the regulation of the *tet* promoter), demonstrating WT levels of *hly* transcription (Fig. S4 and S3D and E). Taken together, these findings indicated that TcyKLMN, CysK, and CymR play a role in the regulation of L. monocytogenes virulence gene expression.

10.1128/mbio.00448-22.2 FIG S2 L. monocytogenes strain 10403S is auxotrophic to cysteine and methionine. (A and B) Growth of WT L. monocytogenes strain 10403S in minimal defined medium supplemented with different concentrations of either cysteine or methionine. The data are presented as maximal OD_600_ values, corresponding to bacterial yield. The data represent 3 biological replicates. Error bars indicate the standard deviation. (C) Growth of WT L. monocytogenes in minimal defined medium without cysteine supplemented with either 1,000 μg/mL, 2,000 μg/mL, or no methionine. Growth of WT L. monocytogenes in minimal defined medium supplemented with standard concentrations of cysteine (100 μg/mL) is shown as a control. OD_600_ values represent growth. The data represent 3 biological replicates. Error bars indicate the standard deviation. (D) Growth of WT L. monocytogenes in minimal defined medium without methionine, supplemented with either 1,000 μg/mL, 2,000 μg/mL, or no cysteine. Growth of WT L. monocytogenes in minimal defined medium supplemented with standard concentrations of methionine (100 μg/mL) is shown as a control. OD_600_ values represent growth. The data represent 3 biological replicates. Error bars indicate the standard deviation. Download FIG S2, PDF file, 0.5 MB. Copyright © 2022 Brenner et al. 2022 Brenner et al. https://creativecommons.org/licenses/by/4.0/ This content is distributed under the terms of the Creative Commons Attribution 4.0 International license.

10.1128/mbio.00448-22.3 FIG S3 Growth of deletion mutants generated in this study in the rich medium BHI. (A to J) WT bacteria and indicated strains were grown in BHI. OD_600_ values represent growth. The data represent 3 biological replicates. Error bars indicate the standard deviation. Download FIG S3, PDF file, 0.6 MB. Copyright © 2022 Brenner et al. 2022 Brenner et al. https://creativecommons.org/licenses/by/4.0/ This content is distributed under the terms of the Creative Commons Attribution 4.0 International license.

10.1128/mbio.00448-22.4 FIG S4 Complementation experiments of Δ*tcyK* and Δ*cysK* mutants. qRT-PCR analysis of the *hly* transcription level in WT L. monocytogenes, Δ*tcyK*, and Δ*cysK* mutants and their respective complementation strains harboring a copy of the *tcyK* or *cysK* gene under the *tet* promoter on the pPL2 plasmid, grown in LBMM. mRNA levels were normalized to *rpoD* mRNA and are represented as relative quantity (RQ), relative to the mRNA level in WT bacteria. The data represent at least 2 biological replicates. Error bars indicate standard deviation. Asterisks represent *P* values (*, *P* < 0.05; **, *P* < 0.01; ***, *P* < 0.001; n.s., nonsignificant) calculated by Student’s *t* test. *P* values represent a comparison to the WT sample unless indicated otherwise. Download FIG S4, PDF file, 0.5 MB. Copyright © 2022 Brenner et al. 2022 Brenner et al. https://creativecommons.org/licenses/by/4.0/ This content is distributed under the terms of the Creative Commons Attribution 4.0 International license.

### TcyKLMN is a cystine/cysteine transporter.

**FIG 2:**
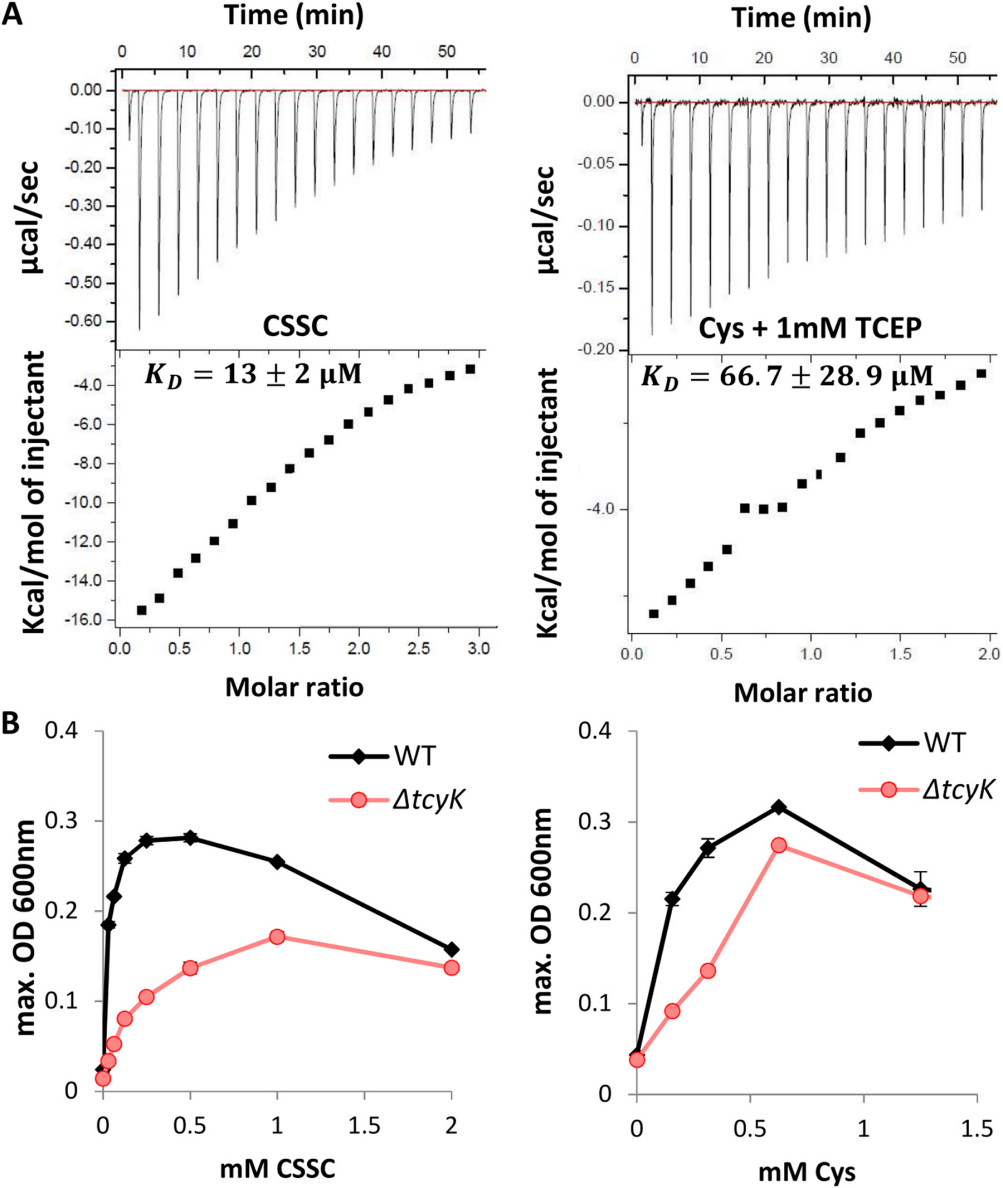
TcyKLMN is a cystine/cysteine transporter. (A) Isothermal titration calorimetry (ITC) analysis, showing binding of TcyK to cystine (CSSC) or cysteine (Cys). Shown are the consecutive injections of 2-μL aliquots from a 200-μM Cys/CSSC solution into 200 μL of 20 μM TcyK. TCEP was used as a reducing agent when indicated. The upper panels show the calorimetric titration, and the lower panels display the integrated injection heat derived from the titrations, for which the best-fit curve was used to calculate the *K_D_*. The experiments were repeated independently 3 times (see also Fig. S5 for two additional biological repeats), and the *K_D_* value is presented as the mean ± standard deviation of these 3 experiments. (B) Growth of WT L. monocytogenes and Δ*tcyK* bacteria grown in minimal defined medium supplemented with different concentrations of cysteine (Cys) or cystine (CSSC) as the sole cysteine source. The data are presented as maximal OD_600_ values and corresponds to bacterial yield. The data represent 2 biological replicates. The experiment was repeated independently twice, with a total of 4 biological replicates. Error bars indicate the standard deviation.

The transport specificity of ABC transporters that function as importers is dictated almost exclusively by the binding specificity of their cognate substrate binding proteins (SBP). Therefore, to determine the substrate specificity of L. monocytogenes TcyKLMN, the SBP of the system, TcyK (containing only amino acids 38 to 286, i.e., without the membrane-anchoring domain), was cloned, overexpressed in Escherichia coli and purified to near homogeneity (Fig. S5A, B). We then used isothermal titration calorimetry (ITC) to measure the binding of different substrates to TcyK. We found that TcyK binds cystine (CSSC, the reduced form of cysteine) with a dissociation constant (*K_D_*) of ∼13 μM, and l-cysteine, with a *K_D_* of ∼66.7 μM (Fig. 2A and Fig. S5C and D). We also tested TcyK for binding of the following amino acids: Val, Leu, Ile, Met, Gln, His, Glu, Thr, and Ser; however, none of these were found to be recognized by TcyK (data shown only for Val, Leu, Ile, and Met; Fig. S6A). These results indicated that TcyK preferentially binds cysteine and cystine. The binding specificity of TcyK was further validated using a tryptophan fluorescence spectroscopy assay, demonstrating TcyK binding of cystine, and not of Val, Leu, Ile, or Met (Fig. S6B). To corroborate these findings, we performed growth experiments of Δ*tcyK* and WT bacteria in minimal defined medium containing either cysteine or CSSC as the sole source. Since L. monocytogenes is auxotrophic to cysteine, bacteria with reduced cysteine-import activity should display a growth disadvantage. Indeed, as shown in Fig. 2B, when either CSSC or cysteine was added at limited concentrations (<0.5 mM), the growth of Δ*tcyK* was significantly attenuated relative to that of WT bacteria. These experiments suggest that L. monocytogenes TcyKLMN imports both CSSC and cysteine.

10.1128/mbio.00448-22.5 FIG S5 Purification of a short variant of TcyK and its binding to cystine and cysteine. (A) Coomassie staining of SDS-PAGE of Ni-NTA affinity purification of a His-tagged short TcyK (amino acids 38 to 286). Lane 1, molecular weight marker (in kDa); lane 2 total protein extract; lane 3 column-unbound fraction; lane 4, wash with 60 mM of imidazole; lanes 5 to 11, fractions eluted using a linear gradient of 60 to 250 mM imidazole. (B) Analysis of the purified protein by size exclusion chromatography; 80 μL of a 0.8 mg/mL protein solution was injected on a Superdex 75 10/300 GL column (GE Healthcare). (C and D) Isothermal titration calorimetry (ITC) analysis, measuring the binding of TcyK to cystine (CSSC) (C) or cysteine (Cys) (D) (two biological repeats are shown for each). Shown are the consecutive injections of 2-μL aliquots from a 200 μM Cys or CSSC solution into 200 μL of 20 μM TcyK. TCEP was used as a reducing agent when indicated. The upper panels show the calorimetric titration, and the lower panels display the integrated injection heat derived from the titrations, for which the best-fit curve was used to calculate the *K_D_*. Additional data for Fig. 2A. Download FIG S5, PDF file, 0.8 MB. Copyright © 2022 Brenner et al. 2022 Brenner et al. https://creativecommons.org/licenses/by/4.0/ This content is distributed under the terms of the Creative Commons Attribution 4.0 International license.

10.1128/mbio.00448-22.6 FIG S6 TcyK does not bind BCAA and methionine. (A) Isothermal titration calorimetry (ITC) analysis, showing the lack of binding of BCAA and methionine. Shown are the consecutive injections of 2-μL aliquots from a 200 μM amino acid stock solution into 200 μL of 20 μM TcyK. (B) Tryptophan fluorescence spectroscopy assay of TcyK in the presence of BCAA and methionine. Shown is the change in the fluorescence (excitation 274 nm, emission 324 nm) of TcyK (2.5 μM) upon addition of 20 μM of the indicated amino acids. The results are the mean (*n* = 3) ± standard deviation of the mean. Download FIG S6, PDF file, 0.5 MB. Copyright © 2022 Brenner et al. 2022 Brenner et al. https://creativecommons.org/licenses/by/4.0/ This content is distributed under the terms of the Creative Commons Attribution 4.0 International license.

### TcyKLMN is negatively regulated by CodY, CysK, and CymR.

**FIG 3:**
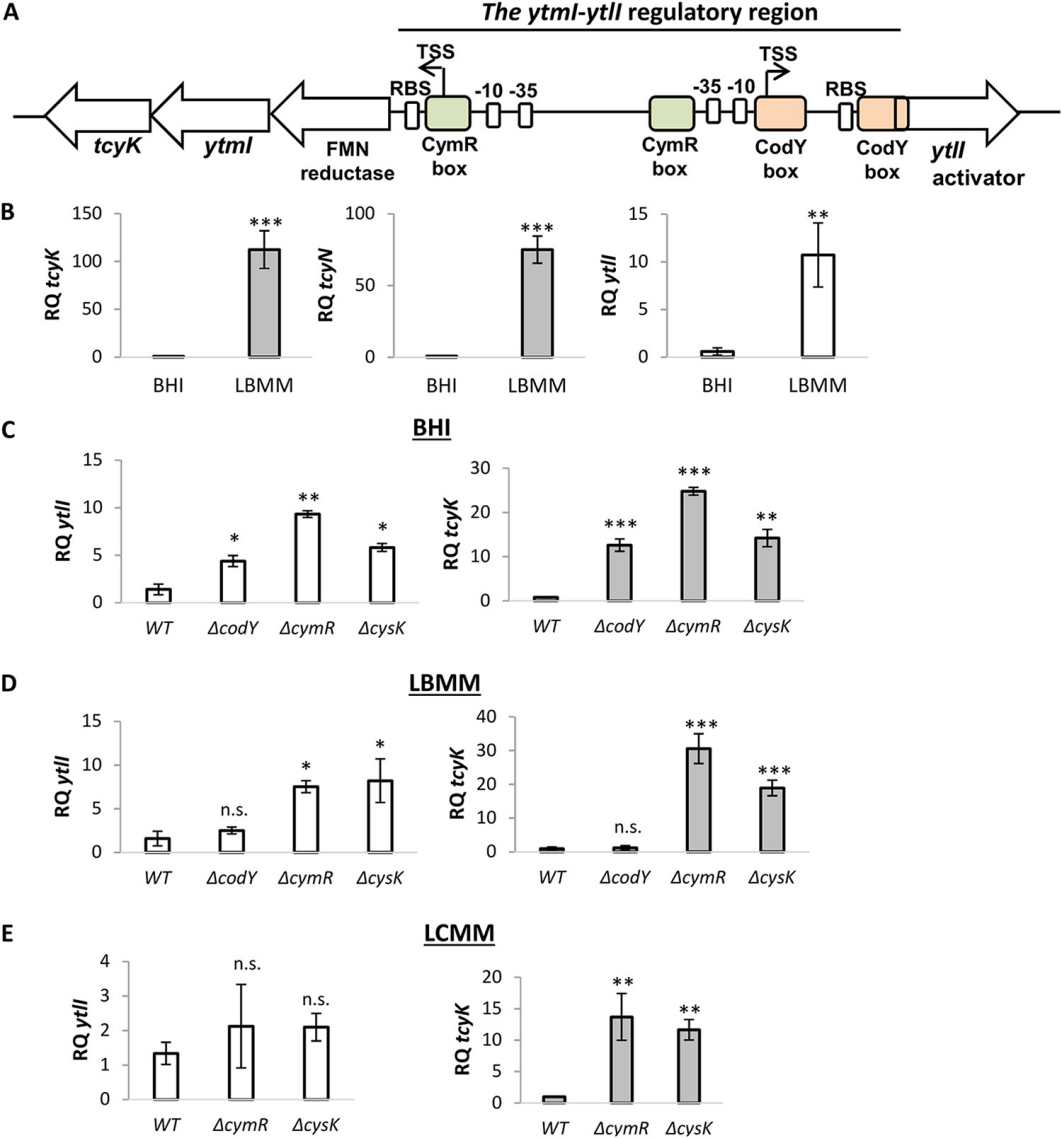
The TcyKLMN transporter is negatively regulated by CodY, CysK, and CymR. (A) A schematic representation of the *ytmI-ytlI* regulatory region. Putative CymR binding sites were identified using the motif of *Bacillus.* Putative CodY binding sites were identified using the motif of Gram-positive bacteria. Promoters (−10 and −35) were identified using BPROM. TSS denotes the transcription start sites, either identified by Wurtzel et al. (64) or predicted based on the promoter sequence. (B) qRT-PCR analysis of *tcyK*, *tcyN*, and *ytlI* transcription level in WT bacteria grown in rich medium (BHI) and in LBMM. mRNA levels were normalized to *rpoD* mRNA and are represented as relative quantity (RQ), relative to the mRNA level in WT bacteria grown in BHI. The data represent 3 biological replicates. Error bars indicate the standard deviation. Asterisks represent *P* values (*, *P* < 0.05; **, *P* < 0.01; ***, *P* < 0.001) calculated by Student’s *t* test. *P* values represent a comparison to the BHI sample. (C to E) qRT-PCR analysis of *tcyK* and *ytlI* transcription level in the indicated mutants grown in rich medium (BHI) and minimal defined medium supplemented with a low concentration of either branched-chain amino acids (LBMM) or cysteine (LCMM). mRNA levels were normalized to *rpoD* mRNA and are represented as relative quantity (RQ), relative to the mRNA level in WT bacteria grown in the indicated medium. The data represent 3 biological replicates. Error bars indicate the standard deviation. Asterisks represent *P* values (*, *P* < 0.05; **, *P* < 0.01; ***, *P* < 0.001; n.s., nonsignificant) calculated by Student’s *t* test. *P* values represent a comparison to the respective WT sample, unless indicated otherwise.

Since the role of TcyKLMN in virulence gene expression was identified under low BCAA, we next investigated its expression level under this condition. To this end, we compared the transcription levels of *tcyK*, *tcyN*, and *ytlI* in WT bacteria grown in LBMM versus BHI. The results demonstrated that the transporter is highly expressed in LBMM (∼100-fold in comparison to BHI, shown by the transcription level of *tcyK* and *tcyN*) and that the YtlI activator gene is also upregulated (∼10-fold) (Fig. 3B). Examining the *ytmI*-*ytlI* intergenic region, we identified two putative CodY binding sites upstream to the *ytlI* gene (also identified in references 22 and 40), suggesting that CodY indirectly regulates TcyKLMN via regulation of the YtlI activator (Fig. 3A). To test this hypothesis, we analyzed the transcription levels of *ytlI* and *tcyK* in WT and Δ*codY* bacteria grown in BHI and LBMM, using qRT-PCR. The experiments confirmed that under nutrient-rich conditions (i.e., in BHI) CodY represses the *ytlI* gene, and consequently *tcyK* (Fig. 3C), and further demonstrated that this repression is relieved when BCAA are limited (i.e., in LBMM) (Fig. 3D). We next investigated the role of CysK and CymR in the regulation of TcyKLMN (i.e., the *ytmI* operon). Of note, since L. monocytogenes does not assimilate sulfate to sulfide, we assumed that CysK acts as a CymR corepressor and not as an *O*-acetylserine thiol-lyase, a function that requires sulfide (Fig. S1B). As mentioned, in B. subtilis it was shown that CysK and CymR form a complex that represses the TcyJKLMN transporter genes (35). Based on the B. subtilis CymR binding motif (38), we identified two putative CymR binding sites in the *ytmI*-*ytlI* regulatory region of L. monocytogenes, one upstream of the *ytlI* gene and the other upstream of the *ytmI* operon (Fig. 3A). To examine the potential regulation of this gene locus by CymR and CysK, we first analyzed the transcription levels of *ytlI* and *tcyK* in Δ*cymR* and Δ*cysK* mutants in comparison to WT bacteria grown in BHI and LBMM. The data clearly demonstrated that CymR and CysK act as repressors, downregulating the transcription of *ytlI* and the *ytmI* operon under both BHI and LBMM conditions (Fig. 3C and D). Since in B. subtilis it was shown that CysK and CymR respond to the availability of cysteine, we repeated this experiment using a minimal defined medium containing a low concentration of cysteine (LCMM, containing 0.08 mM cysteine). Interestingly, the data demonstrated that under these conditions, the repression of *ytlI* by CymR and CysK was fully relieved, whereas *tcyK* (representative of the transporter genes) was still repressed by these proteins (∼15-fold) (Fig. 3E). These findings established that TcyKLMN is negatively regulated, directly or indirectly, by at least three factors, CodY, CymR, and CysK, which collectively respond to changes in BCAA and cysteine levels and possibly to additional metabolites that affect CymR and CysK. Taken together, the data indicated that under nutrient-rich conditions, all three factors repress the *ytmI* operon, whereas under low BCAA conditions, the operon is upregulated, albeit not to its full capacity, as CymR and CysK still downregulate its transcription to some extent. These findings imply a complex regulation of TcyKLMN in response to different metabolic or environmental cues.

**FIG 4:**
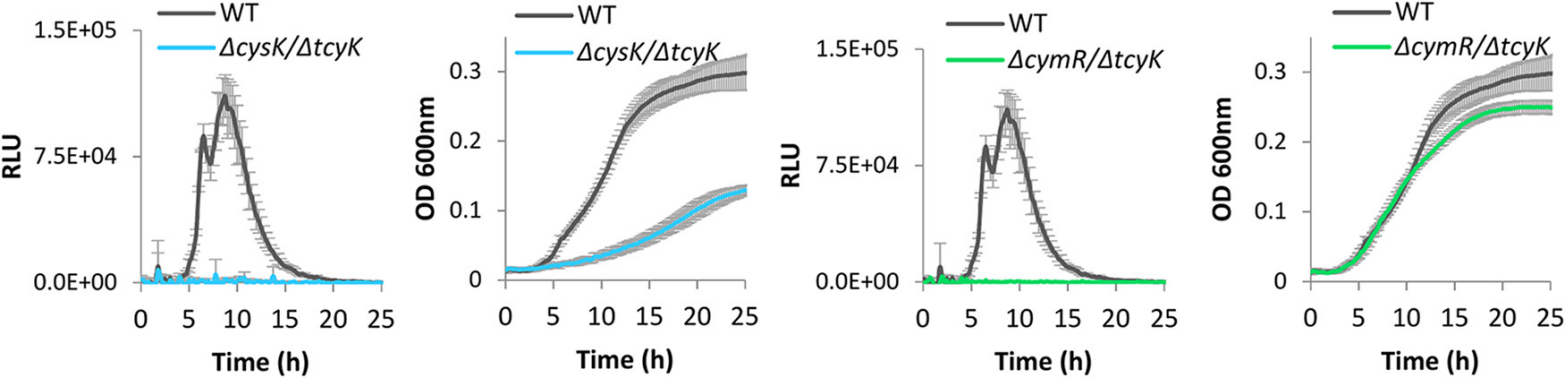
Enhanced expression of virulence genes in Δ*cymR* and Δ*cysK* is TcyKLMN dependent. Luminescence of the pPL2 *Phly-lux* reporter system (left) and growth (right) of Δ*cysK*/Δ*tcyK* and Δ*cymR*/Δ*tcyK* bacteria grown in 96-well plates in minimal defined medium supplemented with a low concentration of branched-chain amino acids (LBMM). Relative luminescence units (RLU) represent luminescence values normalized to the respective OD_600_ value. OD_600_ nm values represent growth. The data represent 3 biological replicates. Error bars indicate the standard deviation.

In light of these observations, we next examined whether the increased expression of the virulence genes in Δ*cymR* and Δ*cysK* is due to the increased expression of TcyKLMN. For this purpose, we generated double deletion mutants, lacking either *cysK* or *cymR* and *tcyK* (Δ*cysK*/Δ*tcyK* and Δ*cymR*/Δ*tcyK*, respectively) and analyzed their *Phly*-*lux* luminescence profiles during growth in LBMM. Notably, both mutants failed to activate *Phly*-*lux* expression, similarly to Δ*tcyK*, indicating that TcyKLMN itself plays a role in the activation of the virulence genes under low BCAA (Fig. 4, in comparison to Fig. 1D and F. Growth in BHI is shown in Fig. S3F and G).

### Cysteine import via TcyKLMN drives glutathione biosynthesis, which triggers the induction of the virulence genes.

**FIG 5:**
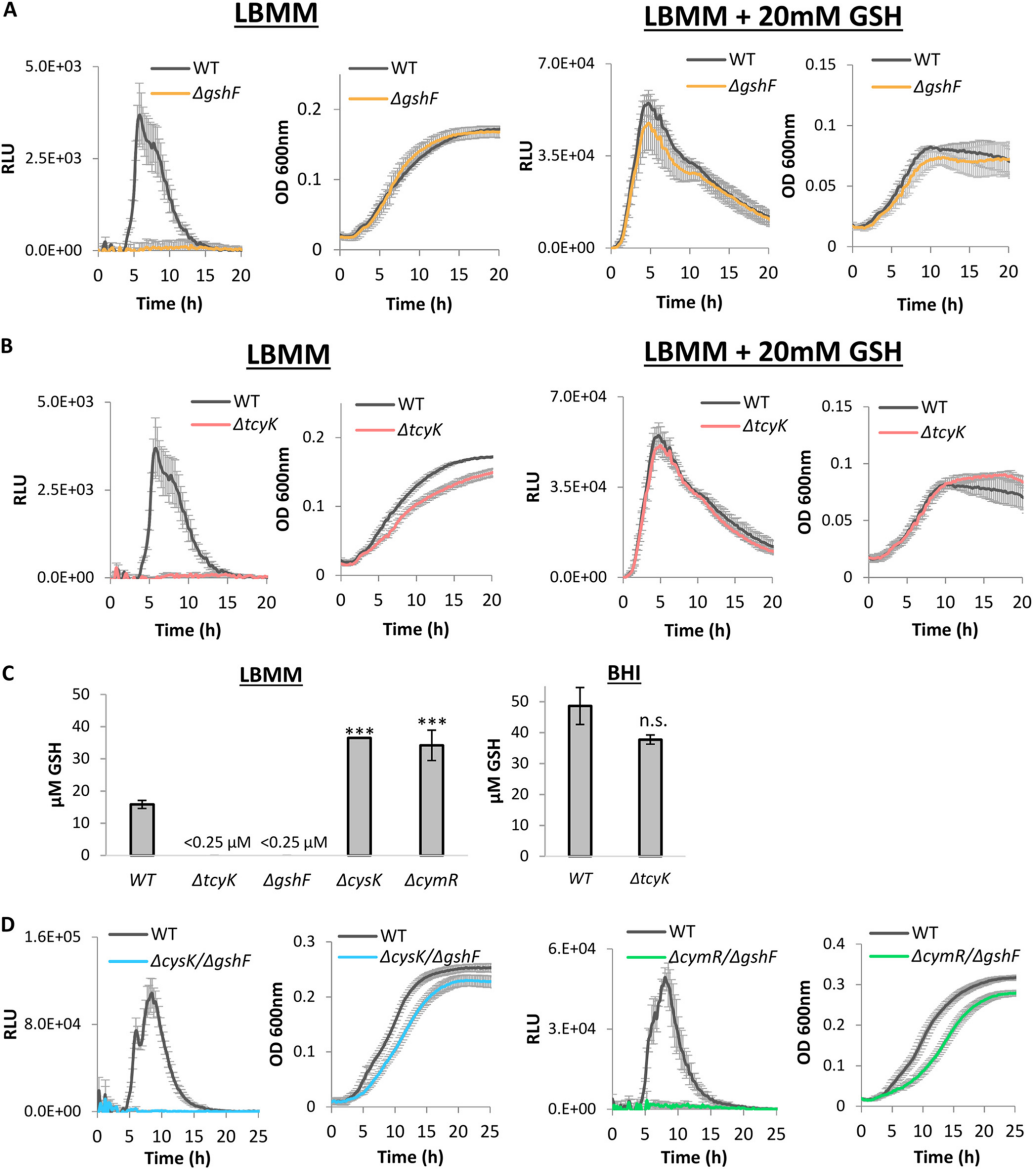
The TcyKLMN transporter plays a role in glutathione biosynthesis. (A and B) Luminescence of the pPL2 *Phly-lux* reporter system (left) and growth (right) of Δ*gshF* and Δ*tcyK* bacteria grown in 96-well plates in minimal defined medium with a low concentration of branched-chain amino acids (LBMM). When indicated, 20 mM reduced glutathione was supplemented to LBMM. Relative luminescence units (RLU) represent luminescence values normalized to the respective OD_600_ value. OD_600_ values represent growth. The data represent 3 biological replicates. Error bars indicate the standard deviation. (C) Internal glutathione level during growth in LBMM (left panel) or BHI (right panel) in indicated bacteria. The data represent 3 biological replicates. Error bars indicate the standard deviation. Asterisks represent *P* values (***, *P* < 0.001; n.s., nonsignificant) calculated by Student’s *t* test. *P* values represent a comparison to the WT sample. (D) Luminescence of the pPL2 *Phly-lux* reporter system (left) and growth (right) of Δ*cysK*/Δ*gshF* and Δ*cymR*/Δ*gshF* bacteria grown in LBMM. Relative luminescence units (RLU) represent luminescence values normalized to the respective OD_600_ value. OD_600_ values represent growth. The data represent 3 biological replicates. Error bars indicate the standard deviation. The plots of Δ*tcyK* are shown for comparison and are derived from the same experiment presented in Fig. 1. The data of WT L. monocytogenes used in panels A and B are the same, as these strains were grown together in the same 96-well plate. The results were separated for better visualization.

Having discovered that in LBMM, TcyKLMN is required for the induction of the virulence genes and not for bacterial growth (Fig. 1E), we hypothesized that its role in cysteine import may directly feed into glutathione biosynthesis, which in turn, stimulates the activity of PrfA. This would account for the failure of all the tested transporter mutants (i.e., Δ*tcyK*, *tcyN*::*Tn*, Δ*cymR*/Δ*tcyK*, and Δ*cysK*/Δ*tcyK*) to express the virulence genes in LBMM. To test this hypothesis, we first constructed a mutant deleted of the glutathione synthase gene (Δ*gshF*) and examined its impact on the activation of virulence genes in LBMM using the *Phly-lux* reporter system. As shown in Fig. 5A, Δ*gshF* displayed practically zero expression of the *lux* genes (similarly to Δ*tcyK*); however this phenotype was fully restored to WT levels by the exogenous addition of GSH (20 mM). These observations indicated that glutathione biosynthesis is absolutely required for the activation of the virulence genes in LBMM. Similarly, the transcription of virulence genes in Δ*tcyK* was restored by the addition of GSH; Δ*tcyK* bacteria supplemented with 20 mM GSH exhibited a luminescence profile that was similar to that of WT bacteria, raising the hypothesis that TcyKLMN is involved in glutathione biosynthesis (Fig. 5B). To directly examine this hypothesis, we measured the total concentration of glutathione (including reduced and oxidized forms, GSH + GSSG) in Δ*tcyK* bacteria in comparison to WT L. monocytogenes, using Δ*gshF* as a control. Remarkably, the internal glutathione concentration of Δ*tcyK* was as low as that measured in Δ*gshF*, below the detection level of the glutathione assay kit (Fig. 5C), indicating that TcyKLMN plays a role in glutathione biosynthesis, most likely by supplying the rate-limiting precursor, cysteine.

**FIG 6:**
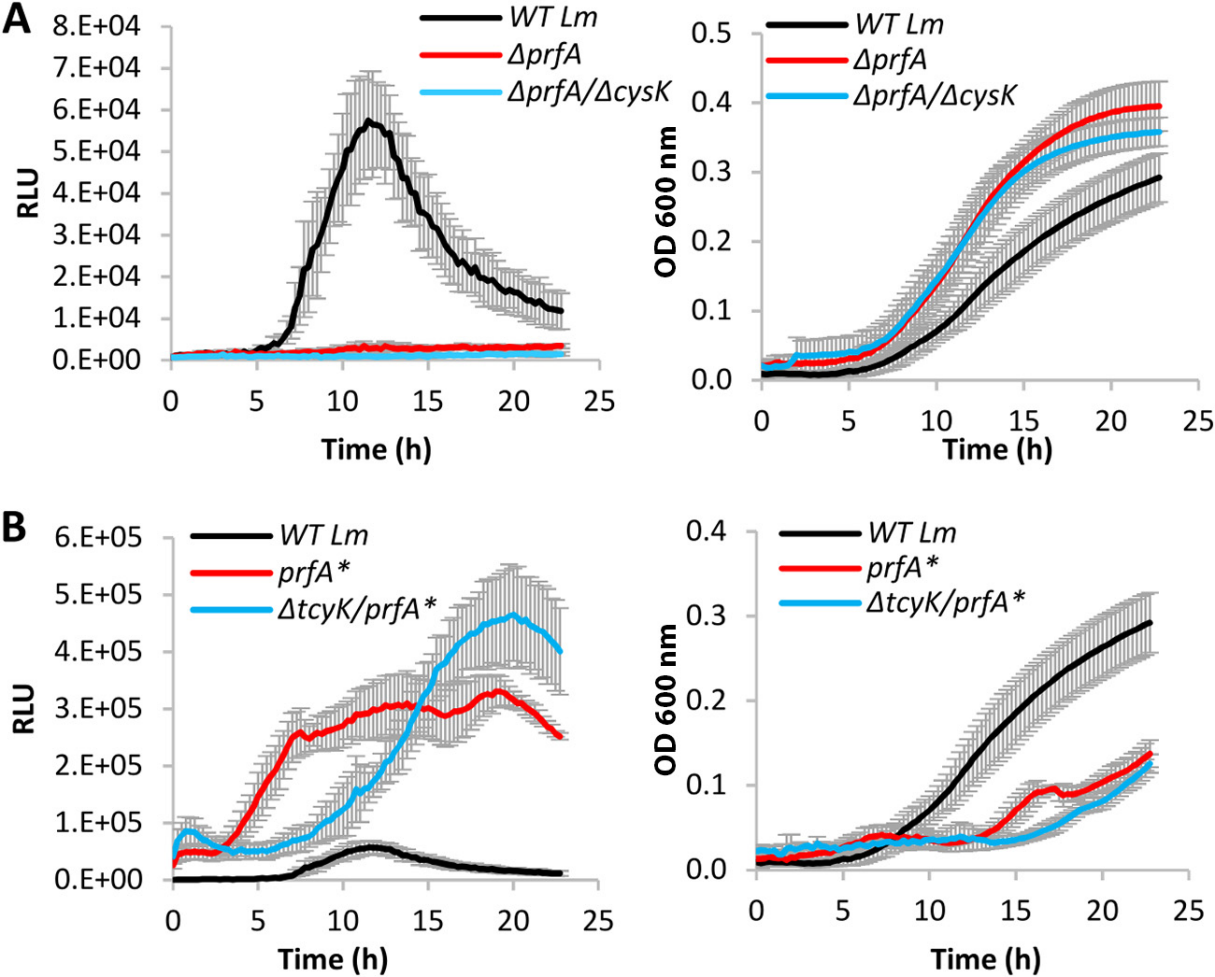
Cysteine uptake by TcyKLMN feeds into PrfA activation. Luminescence profiles (left) and growth (right) of Δ*prfA*/Δ*cysK* and ΔtcyK/prfA* bacteria carrying the pPL2 *Phly-lux* reporter system, grown in minimal defined medium supplemented with a low concentration of branched-chain amino acids (LBMM), compared to WT L. monocytogenes, Δ*prfA*, and prfA* bacteria. Relative luminescence units (RLU) represent luminescence values normalized to the respective OD_600_ value. OD_600_ values represent growth. The data represent 3 biological replicates. Error bars indicate the standard deviation. The data of WT L. monocytogenes used in panels A and B are the same, as these strains were grown together in the same 96-well plate. The results were separated for better visualization.

In light of these findings, we next examined whether the enhanced expression of the virulence genes in Δ*cysK* and Δ*cymR* is a result of increased production of glutathione, as these strains highly express TcyKLMN and hence presumably import more cysteine for glutathione synthesis. Notably, internal measurements of glutathione in Δ*cysK* and Δ*cymR* bacteria demonstrated a 2-fold increase in the amount of glutathione in comparison to WT bacteria, supporting the hypothesis that higher cysteine uptake can increase glutathione biosynthesis (Fig. 5C). Moreover, these observations demonstrated that cysteine import by TcyKLMN feeds into glutathione biosynthesis and further confirmed that cysteine is indeed the rate-limiting substrate of GshF. As a control, we measured GSH internal levels of Δ*tcyK* and WT bacteria grown in the rich medium BHI and found that they are the same (Fig. 5C). These findings indicated that TcyKLMN is not involved in cysteine uptake under nutrient-rich conditions, in line with the observation that this transporter is repressed under these conditions (Fig. 3B). To evaluate whether the enhanced virulence gene expression in Δ*cysK* and Δ*cymR* relies on glutathione biosynthesis, we combined these mutants with a deletion of *gshF* (generating the double deletion mutants Δ*cysK*/Δ*gshF* and Δ*cymR*/Δ*gshF*) and examined virulence gene expression in LBMM, using the *lux* reporter system. As shown in Fig. 5D, these mutants failed to express the *lux* genes, confirming that glutathione biosynthesis is absolutely required for virulence gene expression. Of note, these mutants, as well as Δ*gshF*, grew like WT bacteria in both BHI and LBMM, overall demonstrating that glutathione itself is not essential for growth under these conditions (Fig. 5A and D and Fig. S3H to J). Finally, we examined whether CymR, CysK, and CodY regulate the transcription of *gshF* in BHI and LBMM and found that they do not (Fig. S7), supporting the conclusion that they affect glutathione biosynthesis by regulating the import of cysteine via TcyKLMN. We also examined the possibility that TcyKLMN imports glutathione and found that it does not, as evident from ITC experiments using TcyK and growth experiments using glutathione as a source of cysteine (Fig. S8A and B). Of note, these experiments further demonstrated that L. monocytogenes can use exogenous GSH as a sole source of cysteine (Fig. S8B). Finally, to support the hypothesis that cysteine import by TcyKLMN is required for glutathione biosynthesis, which in turn, activates PrfA, we combined the prfA* mutation (L140F, which renders PrfA constitutively active) with Δ*tcyK* and deleted the *prfA* gene in Δ*cysK* (i.e., generating ΔtcyK/prfA* and Δ*prfA*/Δ*cysK* mutants) and tested the ability of these strains to induce virulence gene expression in LBMM, using the pPL2-P*hly*-*lux* reporter system, in comparison to prfA*, Δ*prfA*, and WT bacteria. As expected, deletion of *prfA* in Δ*cysK* bacteria completely abolished *hly* transcription, whereas expression of PrfA* in Δ*tcyK* bacteria rescued *hly* transcription, overall demonstrating that cysteine import by TcyKLMN feeds into PrfA activation (Fig. 6A and B). Of note was the differential dynamics of *hly* transcription in prfA* bacteria possessing or not possessing TcyK, suggesting that PrfA* is still affected by the presence of glutathione, activated more rapidly. Altogether, these findings demonstrated that when grown in LBMM, TcyKLMN is the main transport system that supplies cysteine for glutathione biosynthesis, which in turn, drives the expression of the virulence genes via the activation of PrfA.

10.1128/mbio.00448-22.7 FIG S7 *gshF* is not transcriptionally regulated by CymR, CysK, and CodY. qRT-PCR analysis of *gshF* transcription level in the indicated mutants grown in rich medium (BHI) and minimal defined medium supplemented with a low concentration of branched-chain amino acids (LBMM). mRNA levels were normalized to *rpoD* mRNA and are represented as relative quantity (RQ), relative to the mRNA level in WT bacteria grown in the indicated medium. The data represent at least 2 biological replicates. Error bars indicate the standard deviation Asterisks represent *P* values (*, *P* < 0.05; **, *P* < 0.01; ***, *P* < 0.001; n.s., nonsignificant) calculated by Student’s *t* test. *P* values represent a comparison to the respective WT sample. Download FIG S7, PDF file, 0.6 MB. Copyright © 2022 Brenner et al. 2022 Brenner et al. https://creativecommons.org/licenses/by/4.0/ This content is distributed under the terms of the Creative Commons Attribution 4.0 International license.

10.1128/mbio.00448-22.8 FIG S8 The TcyKLMN transporter does not transport glutathione. (A) Isothermal titration calorimetry (ITC) analysis, showing no binding of TcyK to reduced glutathione (GSH). Shown are the consecutive injections of 2-μL aliquots from a 200 μM GSH solution into 200 μL of 20 μM TcyK. TCEP was used as a reducing agent. The upper panels show the calorimetric titration, and the lower panels display the integrated injection heat derived from the titrations. The experiment was repeated independently 3 times. (B) Growth of WT L. monocytogenes and Δ*tcyK* bacteria in minimal defined medium supplemented with different concentrations of reduced glutathione (GSH) as the sole source of cysteine. The data are presented as maximal OD_600_ values, corresponding to bacterial yield. The data represent 2 biological replicates. Error bars indicate the standard deviation. Download FIG S8, PDF file, 0.5 MB. Copyright © 2022 Brenner et al. 2022 Brenner et al. https://creativecommons.org/licenses/by/4.0/ This content is distributed under the terms of the Creative Commons Attribution 4.0 International license.

### TcyKLMN contributes to the induction of virulence genes during infection of macrophage cells.

**FIG 7:**
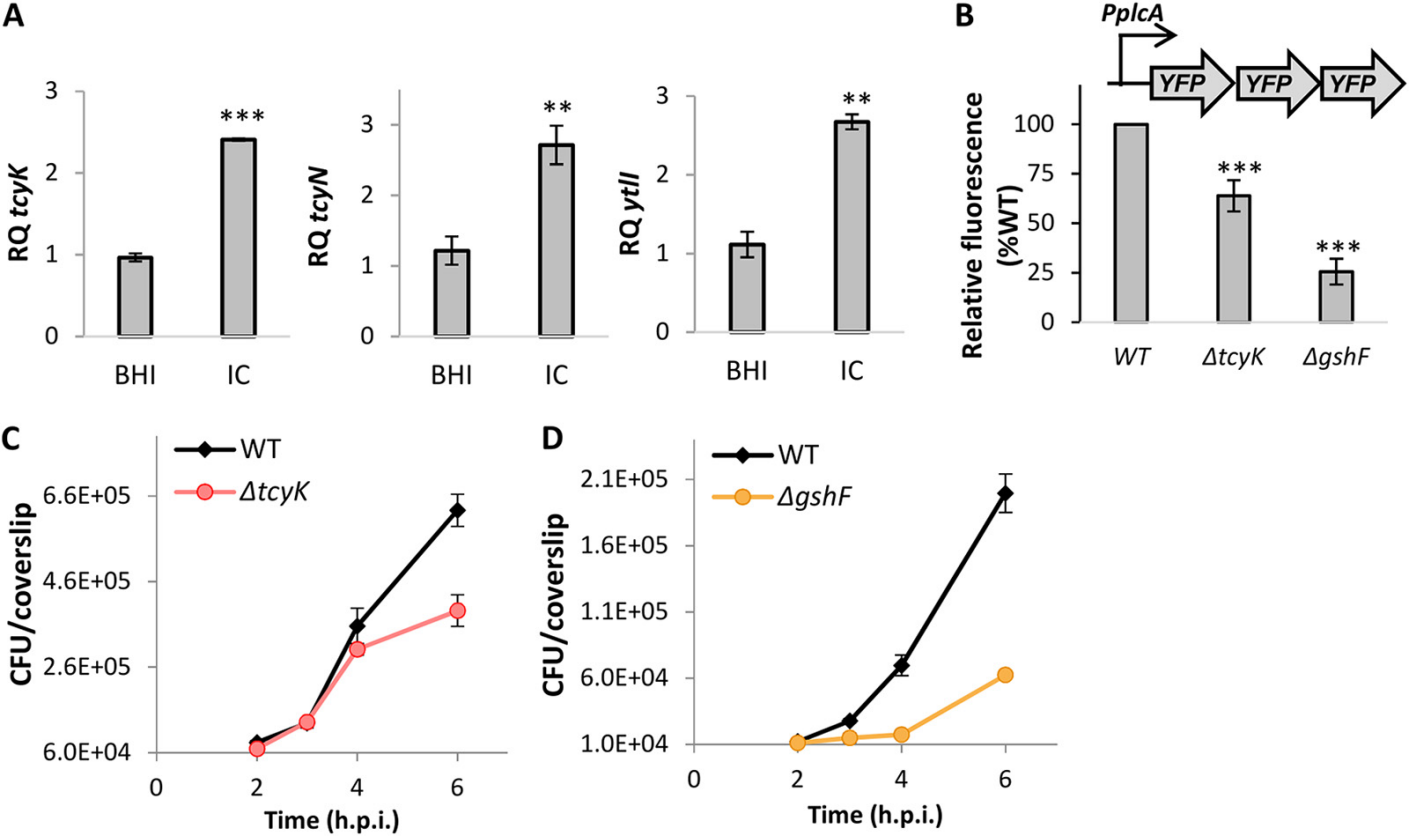
The TcyKLMN transporter promotes L. monocytogenes intracellular growth in macrophage cells. (A) qRT-PCR analysis of *tcyK*, *tcyN*, and *ytlI* transcription levels in WT bacteria grown in rich medium (BHI) or intracellularly in the J774A.1 macrophage cell line (IC). mRNA levels were normalized to *rpoD* mRNA and are represented as relative quantity (RQ), relative to the mRNA level in in the BHI sample. The data represent 3 biological replicates. Error bars indicate the standard deviation. Asterisks represent *P* values (**, = *P* < 0.01; ***, *P* < 0.001) calculated by Student’s *t* test. *P* values represent a comparison to the BHI sample. (B) Intracellular expression of *plcA* as indicated by the fluorescence of YFP expressed under the control of the *plcA* promoter (pPL2-*PplcA-3yfp*) in WT L. monocytogenes and indicated mutants, grown in bone marrow-derived macrophages (BMDM). Fluorescence was measured 3 h postinfection using a florescence microscope. The data represent at least 3 biological replicates. Error bars indicate the standard deviation. Asterisks represent *P* values (**, *P* < 0.01; ***, *P* < 0.001) calculated by Student’s *t* test. *P* values represent a comparison to the WT sample. (C and D) Intracellular growth of Δ*tcyK* and Δ*gshF* mutants in BMDM cells in comparison to WT L. monocytogenes. The experiment was repeated 3 times independently. Since we could not average the data, a representative result is shown here, and two additional biological repeats are shown in Fig. S9. Error bars indicate the standard deviation. h.p.i., hours postinfection.

Finally, given the identified role of TcyKLMN in glutathione biosynthesis, we sought to investigate its impact on L. monocytogenes intracellular growth and virulence gene expression in macrophage cells. We first analyzed the transcription level of *tcyK*, *tcyN*, and *ytlI* in WT bacteria grown intracellularly in macrophage cells in comparison to bacteria grown in BHI. The data indicated a modest induction of TcyKLMN and its activator YtlI within the intracellular niche (∼3-fold) (Fig. 7A). We next evaluated the expression of the virulence gene *plcA* in Δ*tcyK* and WT bacteria grown intracellularly in macrophage cells, using Δ*gshF* as a control. For this purpose, we used a fluorescence-based reporter system that we previously constructed, which relies on the expression of three consecutive *yfp* genes under the regulation of the *plcA* promoter (cloned in the integrative plasmid pPL2, pPL2-P*plcA*-3*yfp*) (33). Using this system, we observed ∼40% reduction in the expression of *plcA* in Δ*tcyK* in comparison to WT bacteria, whereas a more dramatic reduction of ∼75% was observed in Δ*gshF* (Fig. 7B). While these findings demonstrate that TcyKLMN contributes to the induction of the virulence genes during macrophage cell infection, they implied that it is not the sole supplier of cysteine within the intracellular niche, corroborating previous reports demonstrating that L. monocytogenes also utilizes oligopeptides as a source of amino acids, including cysteine, which are imported by the Opp transporter (20, 41). We next examined the intracellular growth of Δ*tcyK* and Δ*gshF* in macrophage cells in comparison to WT bacteria. Notably, the data demonstrated a late growth defect for Δ*tcyK* that was apparent at 6 h postinfection (Fig. 7C and Fig. S9). Interestingly, a similar phenotype was reported for the Opp transporter deletion mutant (Δ*oppDF*), which also exhibited an intracellular growth defect at 6 to 7 h postinfection of macrophage cells (20). Evaluating Δ*gshF* intracellular growth in macrophage cells, we observed a more profound growth defect, starting at 3 h postinfection, indicating that glutathione biosynthesis by L. monocytogenes is critical for intracellular growth and hence has to be accompanied by cysteine uptake (Fig. 7D and Fig. S9). Taken together, the results presented here support the premise that L. monocytogenes uses multiple transport systems to acquire cysteine in the intracellular niche, among them TcyKLMN.

10.1128/mbio.00448-22.9 FIG S9 Intracellular growth of Δ*tcyK* and Δ*gshF* mutants in BMDM cells in comparison to WT L. monocytogenes. The experiment was repeated independently 3 times; two biological repeats for each mutant are shown here, supplementary to Fig. 7C and D. Download FIG S9, PDF file, 0.5 MB. Copyright © 2022 Brenner et al. 2022 Brenner et al. https://creativecommons.org/licenses/by/4.0/ This content is distributed under the terms of the Creative Commons Attribution 4.0 International license.

## DISCUSSION

This study is a direct continuation of our previous reports demonstrating that low availability of BCAA is sensed by L. monocytogenes as a signal to activate the expression of virulence genes. While we established that the metabolic regulator CodY is responsible for the sensing of this signal, we further determined that it directly enhances the transcription of PrfA, thereby promoting virulence gene expression. That said, at the time these studies were conducted, we did not know that glutathione is also involved, through its binding to PrfA and allosterically enhancing its activity (28, 42). Here, we show that the induction of the virulence genes under low-BCAA conditions is entirely dependent on L. monocytogenes’s *de novo* synthesis of glutathione, mediated by GshF. Further, we demonstrate that glutathione biosynthesis under this condition requires the active import of cysteine. As indicated, L. monocytogenes is auxotrophic to cysteine and methionine and hence has to import these amino acids from the environment. To date, two transport systems have been linked to the acquisition of cysteine in L. monocytogenes (as a free amino acid or in peptides), yet their role in supporting L. monocytogenes intracellular growth was not robust, implying that other systems may be involved. Interestingly, studying the regulation of L. monocytogenes virulence gene expression under low BCAA (i.e., in LBMM) led to the discovery of TcyKLMN as a cystine/cysteine importer that facilitates glutathione biosynthesis and PrfA activation. The results demonstrated that TcyKLMN is repressed under nutrient-rich conditions and upregulated when BCAA are limited. This repression was linked to CodY, which we found to repress the activator of the *ytmI* operon, YtlI. As indicated, low BCAA is the same condition under which PrfA expression is upregulated by CodY; therefore, it is possible that the parallel increase in cysteine uptake, and subsequent upregulation of glutathione biosynthesis, may have evolved to cope with the increased levels of PrfA. Altogether, the findings presented here suggest an intriguing synchronization between PrfA expression and glutathione biosynthesis that is mediated by CodY and further indicate that CodY regulation of L. monocytogenes virulence is even more complex than previously considered.

An important observation made in this study was that glutathione levels are largely limited within the bacteria (especially when grown in low-nutrient conditions) and that the availability of cysteine determines its biosynthesis. In line with this conclusion, all the factors that were found to affect cysteine import, i.e., CodY, CymR, CysK, and TcyKLMN, indirectly influenced virulence gene expression. In a way, these findings uncover a new pathway by which L. monocytogenes virulence can be regulated in the mammalian host, responding to changes in cysteine availability. CodY, CymR, and CysK were all found to regulate the expression of TcyKLMN and hence to modulate the expression of the virulence genes. These regulators still repressed TcyKLMN under conditions of low concentrations of BCAA and cysteine, implying that additional metabolic or environmental signals may be involved. In B. subtilis, it was shown that the *ytmI* operon is differentially regulated under disulfide stress and changes in sulfur availability; hence, it is possible that the L. monocytogenes CymR and CysK respond to these conditions (36, 38). Moreover, since glutathione plays a role in redox conditions, it is possible that CymR and CysK respond to these conditions as well. In this respect, it was demonstrated in Staphylococcus aureus that CymR senses oxidative stress via thiolation of its cysteine residue (Cys-25), and hence, the YtmI operon is upregulated in oxidizing environments (43). Taken together, these reports indicate that the YtmI operon is regulated by multiple metabolic and environmental cues. Interestingly, the *ytmI* operon of B. subtilis is independent of CodY regulation, as evident from transcriptome sequencing (RNA-seq) experiments and genome-wide analyses of CodY binding sites (44, 45). It is tempting to speculate that CodY regulation of L. monocytogenes’s TcyKLMN evolved to cope with the pathogenic lifestyle of this bacterium, yet L. monocytogenes and B. subtilis differ greatly in their sulfur, cysteine, and methionine metabolism, which can lead to differences in the regulation of these pathways. Nevertheless, this study revealed an intriguing link between CodY regulation, cysteine import, and glutathione biosynthesis in L. monocytogenes, depicting an additional mechanism to control the expression of the virulence genes.

Here, we showed that the SBP of TcyKLMN (i.e., TcyK) specifically binds cysteine, and to a better extent CSSC, suggesting that it primes the import of these two related substrates. CSSC is the oxidized form of cysteine, containing two cysteine molecules that are linked via a disulfide bond, and hence is expected to be more prevalent in oxidizing environments, such as within phagosomes. Outside the mammalian cells (i.e., in the extracellular milieu), cysteine is considered to be abundant (produced and secreted by the liver), yet it is quickly oxidized and imported into the cells via specialized CSSC transporters, e.g., the CSSC/glutamate antiporter, cXT, which also plays a role in glutathione synthesis (46–49). Interestingly, within the cells, thioredoxin or glutathione are used to reduce the CSSC to cysteine, which is further utilized in protein synthesis. Of note, mammalian cells can further biosynthesize cysteine from methionine, using the transsulfuration pathway, or alternatively, break down glutathione to salvage cysteine (46). In respect to L. monocytogenes’s intracellular lifestyle, it is likely that it encounters CSSC within the phagosome/vacuole environment. It is possible that in nature TcyKLMN plays a role during cell invasion or when L. monocytogenes switches to persistent infection, e.g., when it resides in lysosome-like vacuoles for long periods (50, 51). While we did not identify a phenotype for Δ*tcyK* upon intravenous mouse infections, we observed a late intracellular growth defect during infection of bone marrow-derived macrophage cells. These experiments also demonstrated a reduced transcription level of *plcA* in Δ*tcyK*, supporting the premise that TcyKLMN contributes to the activation of PrfA during L. monocytogenes infection of mammalian cells. Since CSSC and cysteine are relatively limited in the intracellular environment, it is not surprising that L. monocytogenes acquired multiple means to scavenge this essential amino acid. As mentioned, L. monocytogenes exploits peptides as a source of amino acids, and the Opp transporter was shown to import cysteine-containing peptides as a source of cysteine for glutathione biosynthesis (20, 41). The finding that Δ*tcyK* exhibits a 40% reduction in *plcA* transcription, compared to the 75% reduction in Δ*gshF*, supports the conclusion that L. monocytogenes exploits multiple systems to scavenge cysteine.

Reniere et al. suggested that L. monocytogenes also exploits host glutathione (GSH) to activate PrfA (17). However, this mode of PrfA activation was shown to be minor, as Δ*gshF* bacteria hardly activated the virulence genes in the intracellular environment (17), overall indicating that host-derived GSH does not replace the glutathione produced by L. monocytogenes. In this regard, it remains an open question why L. monocytogenes synthesizes GSH and uses it as an intracellular signal, considering its high abundance in the intracellular niche, much more than cysteine. Other bacterial pathogens, such as Burkholderia pseudomallei and Francisella tularensis, were shown to exploit host GSH as an activating signal of virulence gene expression and as a source of cysteine, respectively (52, 53). Intriguingly, *in vitro* experiments in minimal defined medium indicated that L. monocytogenes is capable of importing GSH and uses it both to activate PrfA and as a source of cysteine (Fig. 5 and Fig. S8B) (54). While these findings imply that L. monocytogenes encodes a glutathione transporter, or alternatively, uses a nonspecific system such as di-/tri-peptide transporters to import glutathione, it is not clear why it does not use them in the intracellular niche. Despite its importance, a dedicated glutathione transporter was not identified in Gram-positive bacteria. In Gram-negative bacteria the dipeptide transporter DppBCDF was shown to import glutathione, using the SBP protein GbpA (55). It is of particular interest to decipher the structural and molecular mechanism by which glutathione is imported in Gram-positive bacteria, especially intracellular pathogens such as L. monocytogenes. Interestingly, in the Gram-positive bacterium Streptococcus mutans, it was shown that the CSSC ABC transporter, TcyBC (not found in L. monocytogenes) imports both CSSC and glutathione using two distinct SPB proteins (55). The SBP that binds glutathione (GshT) was found to be encoded elsewhere on the bacterial chromosome, and not near the *tcyBC* genes. While this phenomenon of a shared permease is not new, the discovery that S. mutans holds a distinct SBP that primes glutathione import via another transporter was new, providing early insights into glutathione import in Gram-positive bacteria (56). In this regard, we found that TcyK shares 30% amino-acid sequence identity with the S. mutans GshT, yet our data indicated that it does not bind glutathione.

In summary, this study demonstrated that cysteine import is critical for virulence gene expression in L. monocytogenes. The data imply that multiple metabolic and environmental cues are involved in the regulation of cysteine import and hence affect glutathione biosynthesis, placing this function at the heart of L. monocytogenes patho-metabolism. As different bacterial pathogens acquired different mechanisms to sense the mammalian niche, it is interesting to learn how they exploit host and bacterial metabolites as signals and effectors of virulence.

## MATERIALS AND METHODS

### Ethics statement.

Experimental protocols were approved by the Tel Aviv University Animal Care and Use Committee (01-15-052, 04-13-039) and were in accordance with the Israel Welfare Law (1994) and the National Research Council guide (Guide for the Care and Use of Laboratory Animals 2010).

### Bacterial strains, plasmids, and primers.

Listeria monocytogenes strain 10403S was used as the WT strain and as the parental strain to generate allelic exchange mutant strains (Table S1). E. coli XL-1 blue strain (Stratagene) was used to generate vectors, and E. coli SM-10 strain (57) was used for plasmid conjugation to L. monocytogenes. The plasmids and primers used in this study are listed in Table S1.

10.1128/mbio.00448-22.10 TABLE S1 Strains, plasmids, and primers used in this study. Download Table S1, DOCX file, 0.02 MB. Copyright © 2022 Brenner et al. 2022 Brenner et al. https://creativecommons.org/licenses/by/4.0/ This content is distributed under the terms of the Creative Commons Attribution 4.0 International license.

### Growth conditions.

L. monocytogenes bacteria were grown at 37°C, with agitation, in brain heart infusion (BHI), as a nutrient-rich medium, or in minimal defined medium (MM). MM (phosphate buffer 48.2 mM KH_2_PO_4_ and 1.12 M Na_2_HPO_4_, pH 7, 0.41 mg/mL magnesium sulfate, 10 mg/mL glucose, 100 μg/mL of each amino acids [methionine, arginine, histidine, tryptophan, phenylalanine, cysteine, isoleucine, leucine, and valine], 600 μg/mL glutamine, 0.5 mg/mL biotin, 0.5 mg/mL riboflavin, 20 mg/mL ferric citrate, 1 mg/mL para-aminobenzoic acid, 5 ng/mL lipoic acid, 2.5 mg/mL adenine, 1 mg/mL thiamine, 1 mg/mL pyridoxal, 1 mg/mL calcium pantothenate, and 1 mg/mL nicotinamine) was prepared as described previously (58). For analysis of auxotrophies, L. monocytogenes was grown with 0 to 2,000 μg/mL of either cysteine or methionine or neither, as indicated. For growth under low-BCAA conditions, MM was freshly made with 10-fold less isoleucine, leucine, and valine (resulting in a final concentration of 10 μg/mL) and was named low-BCAA minimal defined medium (LBMM). For growth in limited concentrations of cysteine, MM was freshly made with 10-fold less cysteine (resulting in a final concentration of 10 μg/mL). For growth in glutathione medium, 20 mM reduced glutathione (GSH) was freshly added to LBMM. For growth with cysteine or with either cystine (CSSC) or reduced or oxidized glutathione (GSH or GSSG, respectively) as a cysteine source, MM was prepared without cysteine, and 0 to 2 mM the respective cysteine source was added to the medium.

### Lux reporter assay.

Overnight BHI cultures harboring a *Phly*-*lux* luciferase reporter system (pPL2*-Phly-luxABCDE*) (59) were washed 3 times with phosphate-buffered saline (PBS), adjusted to an optical density at 600 nm (OD_600_) of 0.03 in fresh LBMM or GSH medium, and grown in a 96-well plate. Luminescence and bacterial growth (OD_600_) were measured every 15 min after shaking for 2 min, using a Synergy HT BioTek plate reader at 37°C for 12 to 48 h. Since in each 96-well plate different strains were grown in comparison to WT bacteria, some of the figures show the same WT samples that were grown in that plate. The results were separated for better visualization of the data.

### Bacterial RNA extraction and qRT-PCR.

Bacteria grown in the indicated medium were harvested at mid-logarithmic growth (OD_600_ of ∼0.3). Total RNA was extracted from bacteria using the RNAsnap method (60). Briefly, bacterial pellets were washed with AE buffer (50 mM NaOAc, pH 5.2, 10 mM EDTA) and then resuspended in 95% formamide, 18 mM EDTA, 1% 2-mercaptoethanol, and 0.025% SDS. Bacterial lysis was performed by vortexing extracts with 100 μm zirconia beads (OPS Diagnostics), followed by incubation at 95°C. Nucleic acids were precipitated with ethanol and treated with Turbo-DNase (Ambion), followed by standard phenol extraction. Total RNA (1 μg) was reverse-transcribed to cDNA using qScript (Quanta). qRT-PCR was performed on 10 ng cDNA using FastStart Universal SYBR green master mix (Roche) in a StepOnePlus real-time PCR system (Applied Biosystems). The transcription level of each gene was normalized to that of the reference gene, *rpoD*.

### Analysis of promoters and putative binding sites.

Putative binding sites of transcriptional regulators were identified using the MAST search tool (61), using the CymR motif of *Bacillus* (38) and CodY motif of Gram-positive bacteria (62). Promoters (−10 and −35) were identified using BPROM (63). Transcription start sites (TSS) were either previously identified (64) or manually predicted based on the promoter sequence.

### Intracellular growth in macrophage cells.

Bone marrow-derived macrophages (BMDM) were used for L. monocytogenes infection experiments. The cells were isolated from 8-week-old female C57BL/6 mice (Envigo, Israel) and cultured in BMDM medium (Dulbecco’s modified Eagle medium [DMEM] supplemented with 20% fetal bovine serum, sodium pyruvate [1 mM], l-glutamine [2 mM], β-mercaptoethanol [0.05 mM], and monocyte-colony-stimulating factor [M-CSF, L929-conditioned medium], as described previously [65]). BMDM cells (2 × 10^6^) were seeded in a 60-mm petri dish, on glass coverslips, in 5 mL BMDM medium and incubated overnight (O.N.) in a 37°C, 5% CO_2_ forced-air incubator. L. monocytogenes bacteria (8 × 10^6^) grown O.N. at 30°C without agitation, were used to infect BMDM cells (multiplicity of infection [MOI] of 1). Then, 30 min postinfection, macrophage monolayers were washed and fresh medium was added. Gentamicin was added 1 h postinfection to a final concentration of 5 μg/mL in order to limit the growth of extracellular bacteria. At each time point, three coverslips were transferred to 2 mL sterile water to release the intracellular bacteria. Serial dilutions of the resulting lysate were plated on BHI agar plates, and the CFU were counted after 24 h of incubation at 37°C.

### Intracellular P*plcA*-3*yfp* expression.

WT and mutant strains expressing three consecutive yellow fluorescent proteins (YFP) under the regulation of the *plcA* gene promoter (cloned on the pPL2 integrative plasmid) were used to infect BMDM on 20-mm slides. Then, 3 h postinfection, cells were fixed with 4% vol/vol paraformaldehyde-PBS and permeabilized with 0.05% vol/vol Triton X-100. DNA was stained with DAPI (4′,6-diamidino-2-phenylindole)-containing Vectashield mounting medium (Vector Laboratories, Inc.). Fluorescent images were captured using a Nikon eclips microscope.

### Intracellular gene expression analysis.

RNA was purified from WT L. monocytogenes bacteria intracellularly grown in J774A.1 macrophage cells, as previously described for BMDM macrophages (66). Briefly, three 145-mm dishes were seeded with 2 × 10^7^ cells that were then infected in parallel with 2 × 10^9^ bacteria. Then, 30 min postinfection, cell monolayers were washed twice with PBS to remove unattached bacteria, and fresh medium was added. At 1 h postinfection (hpi), gentamicin (50 μg/mL) was added to limit extracellular bacterial growth. At 6 hpi, the macrophages were lysed with 20 mL cold water, and cell debris and nuclei were removed by centrifugation (800 × *g*, 3 min, 4°C). Released bacteria were quickly collected on 0.45-μm filter membranes (Millipore) using a vacuum apparatus and snap-frozen in liquid nitrogen. Bacteria were recovered from the filters by vortexing the membranes in AE buffer (50 mM sodium acetate, pH 5.2, 10 mM EDTA), and bacterial nucleic acids were extracted using the RNAsnap method (60), followed by ethanol precipitation. An RNeasy mini kit DNase column (Qiagen) was used for DNase treatment.

### Cloning, overexpression, and purification of TcyK.

L. monocytogenes 10403S *tcyK*, encoding the TcyKLMN substrate binding protein, was synthesized and adjusted to the E. coli codon usage (Genescript). The gene was cloned into the pET-19b vector (Novagen) for expression with an N-terminal His-tag. His-tagged TcyK was overexpressed in E. coli BL21-Gold (DE3) (Stratagene) cultured in Terrific Broth (TB) and induced at mid-log phase by addition of 1 mM isopropyl-β-d-1-thiogalactopyranoside (IPTG; 1.5 h, 37°C). Cells were harvested by centrifugation (13,600 × *g*, 20 min, 4°C), and the pellet was stored at −80°C until use. For purification, cells were homogenized in 50 mM Tris-HCl, pH 8, 500 mM NaCl, complete EDTA-free protease inhibitor (Roche), 30 mg mL^−1^ DNase (Worthington), and 1 mM MgCl_2_. The cells were then ruptured by three passages through an EmulsiFlex-C3 homogenizer (Avestin), and the lysate was centrifuged at 34,500 × *g* for 30 min at 4°C. The supernatant was loaded onto a nickel affinity column (HisTrap HP, GE Healthcare) on an AKTA Avant instrument. The protein was eluted using an imidazole gradient, and imidazole was removed from the sample by desalting (HiPrep 26/10, GE Healthcare). Protein purification was monitored by Coomassie staining of SDS-PAGE and size exclusion chromatography (Superdex 75 10/300 GL, GE Healthcare).

### Isothermal titration calorimetry experiments.

Calorimetric measurements were performed with the Microcal iTC200 system (GE Healthcare). Prior to measurements, purified TcyK was dialyzed against three exchanges of 50 mM Tris-HCl, pH 8, and 500 mM NaCl buffer. Stocks of cysteine, CSSC, GSSG, GSH, or control amino acid (Val, Leu, Ile, Met, Gln, His, Glu, Thr, and Ser) were prepared fresh in double-distilled water and diluted to a working concentration using the buffer from the last protein dialysis exchange. Then, 1 mM Tris(2-carboxyethyl)phosphine (TCEP) was added to cysteine and GSH stocks as a reductant agent. Aliquots (2 μL) of 500 μM stocks were added by a rotating syringe to the reaction well containing 200 μL of 50 μM purified TcyK at 25°C. Data-fitting was performed with MicroCal analysis software.

### Tryptophan fluorescence spectroscopy analysis of protein-ligand binding.

Purified TcyK was dialyzed overnight against 1,000-fold of 50 mM Tris-HCl, pH 8, and 500 mM NaCl (3 buffer replacements). Amino acid stocks were prepared fresh in double-distilled water or 1 M HCl (in the case of cystine and isoleucine) and diluted to working concentrations using the dialysis buffer. Proteins were diluted to 2.5 μM in dialysis buffer or to the same final concentration of HCl. Triplicates of 100 μL of TcyK proteins and amino acids were measured using a monochromator-based Tecan M200 plate reader (excitation of 274 nm and emission of 324 nm).

### Glutathione quantification.

The total glutathione (GSH+GSSG) concentration in bacteria was measured as described previously (54). Briefly, bacteria were grown to mid-log phase in LBMM, resuspended in PBS containing 1 mM EDTA, pH 6.5, and lysed by vortexing with 100-μm zirconia beads (OPS Diagnostics). Cold (4°C) lysates were deproteinated with an equal volume of metaphosphoric acid and quantified with a commercial kit (Cayman Chemical) according to the recommendations of the kit manufacturer.
