# Supplementary figures and images for: Listeria monocytogenes TcyKLMN Cystine/Cysteine Transporter Facilitates Glutathione Synthesis and Virulence Gene Expression
Source: mBio. 2022 Apr 18;13(3):e00448-22. doi: 10.1128/mbio.00448-22 (PMC9239247; doi:10.1128/mbio.00448-22)

A

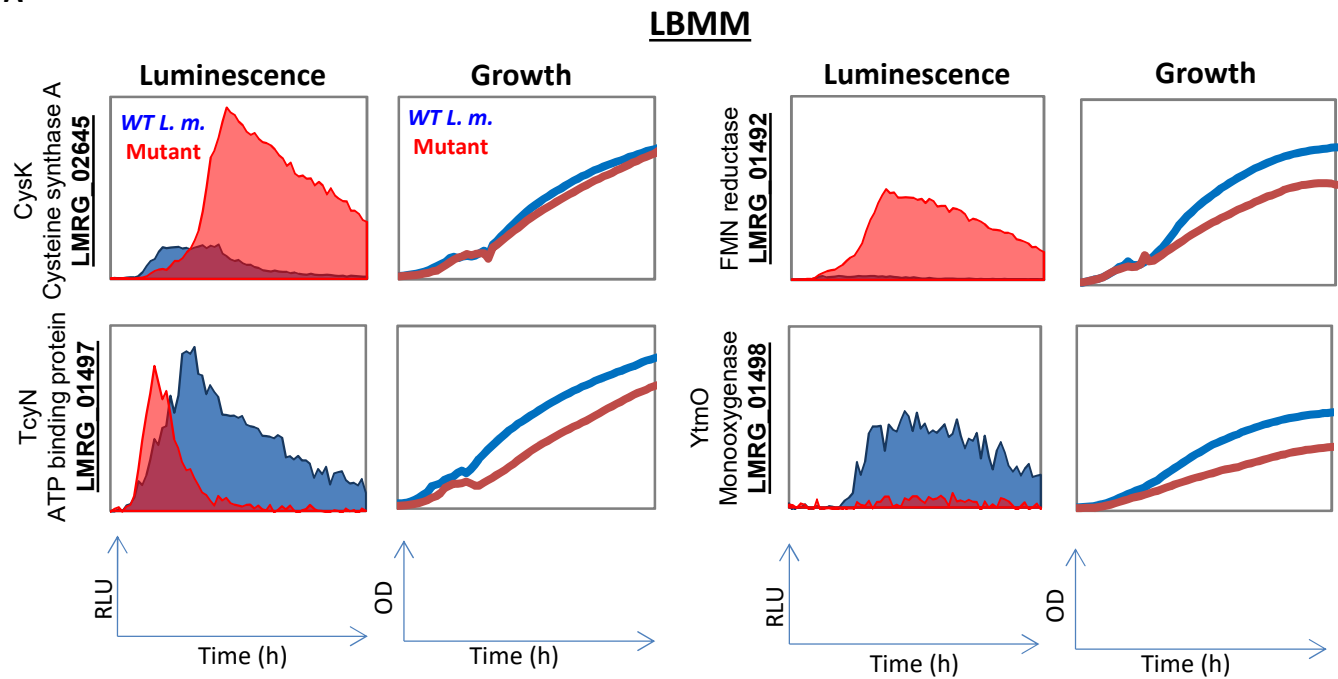

B

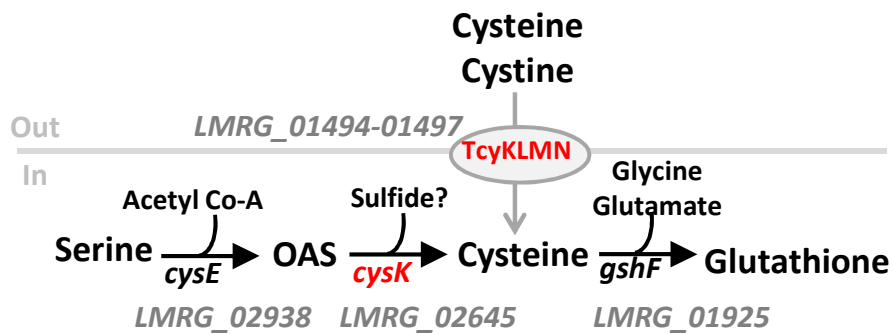

Fig S1

Supplement: FIG S1 [file mbio.00448-22-s0001.pdf]

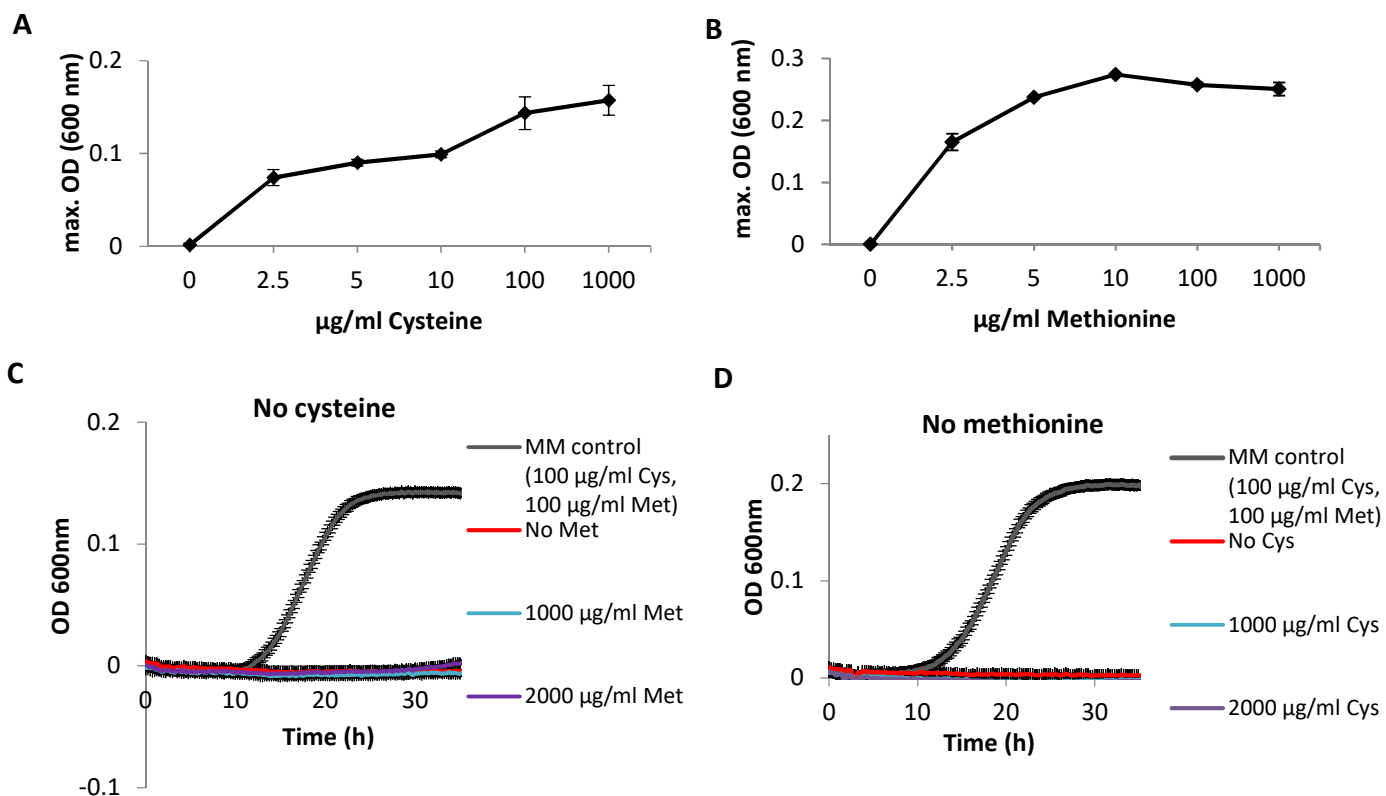

**Fig S2**

Supplement: FIG S2 [file mbio.00448-22-s0002.pdf]

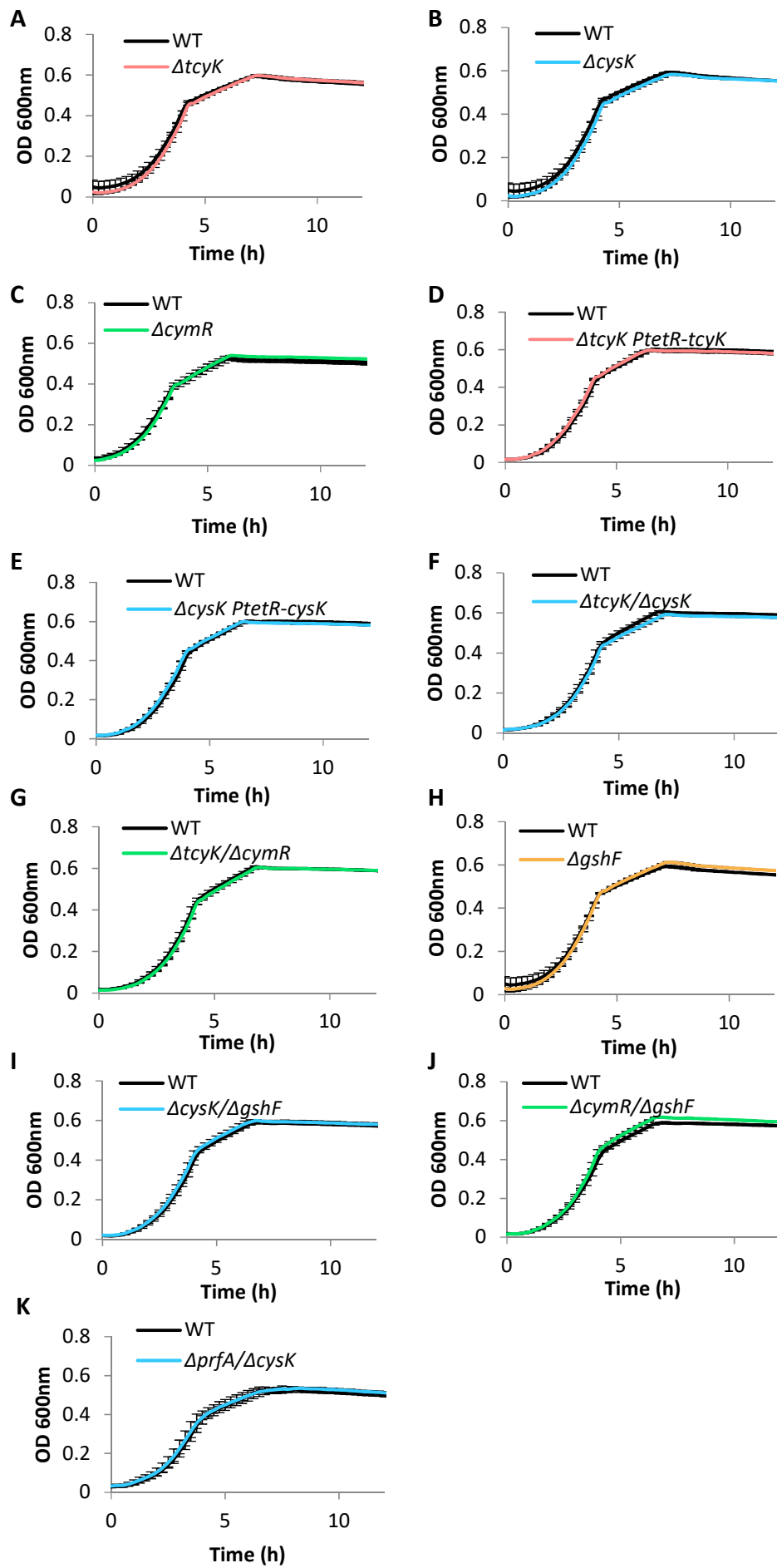

**Fig S3**

Supplement: FIG S3 [file mbio.00448-22-s0003.pdf]

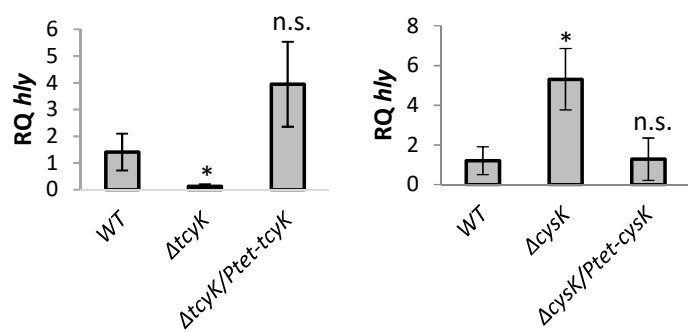

**Fig S4**

Supplement: FIG S4 [file mbio.00448-22-s0004.pdf]

**A**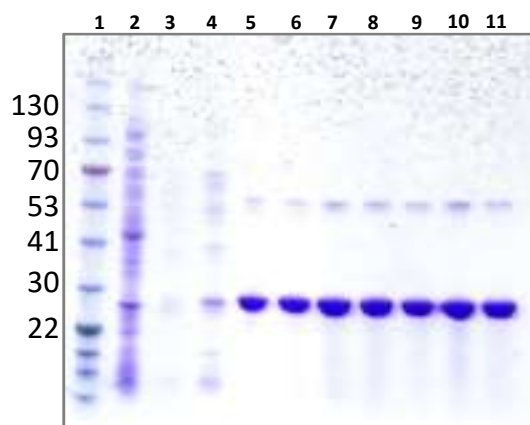**B**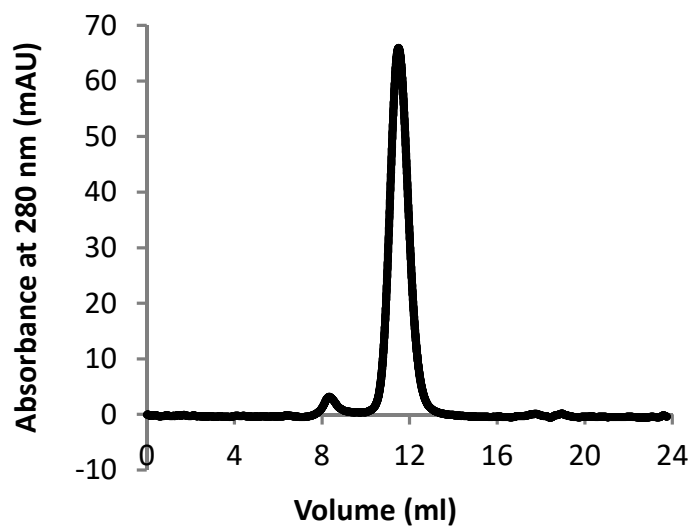**C**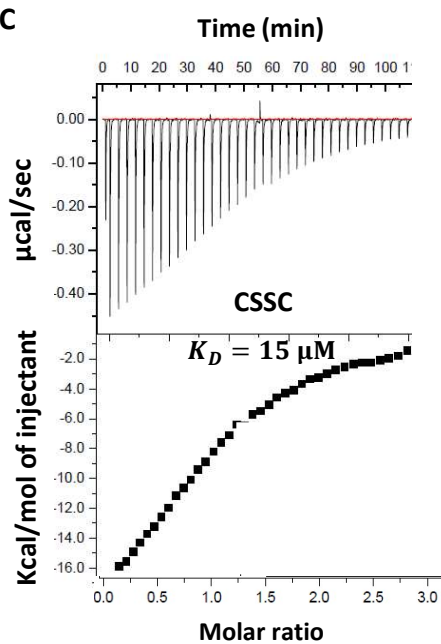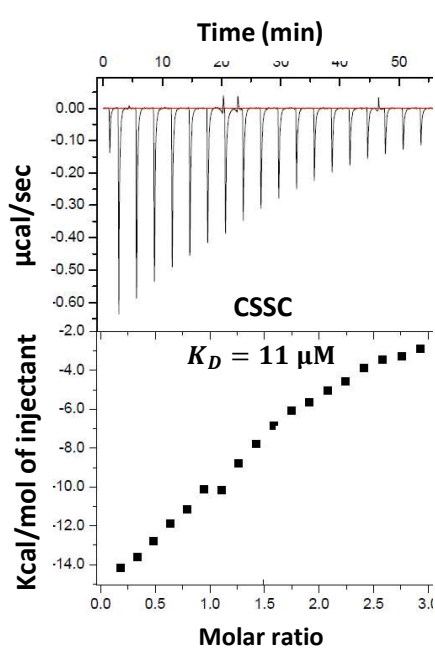**D**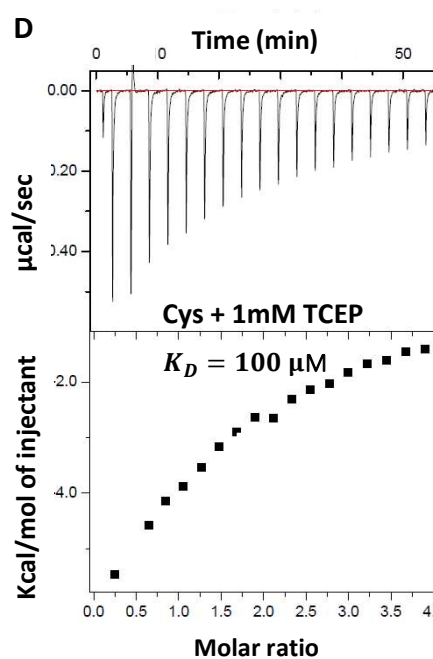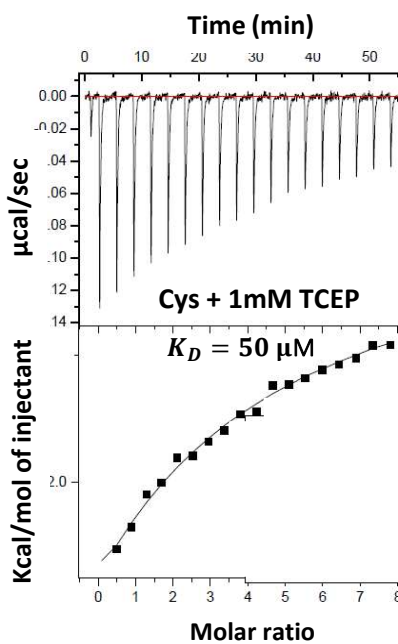**Fig S5**

Supplement: FIG S5 [file mbio.00448-22-s0005.pdf]

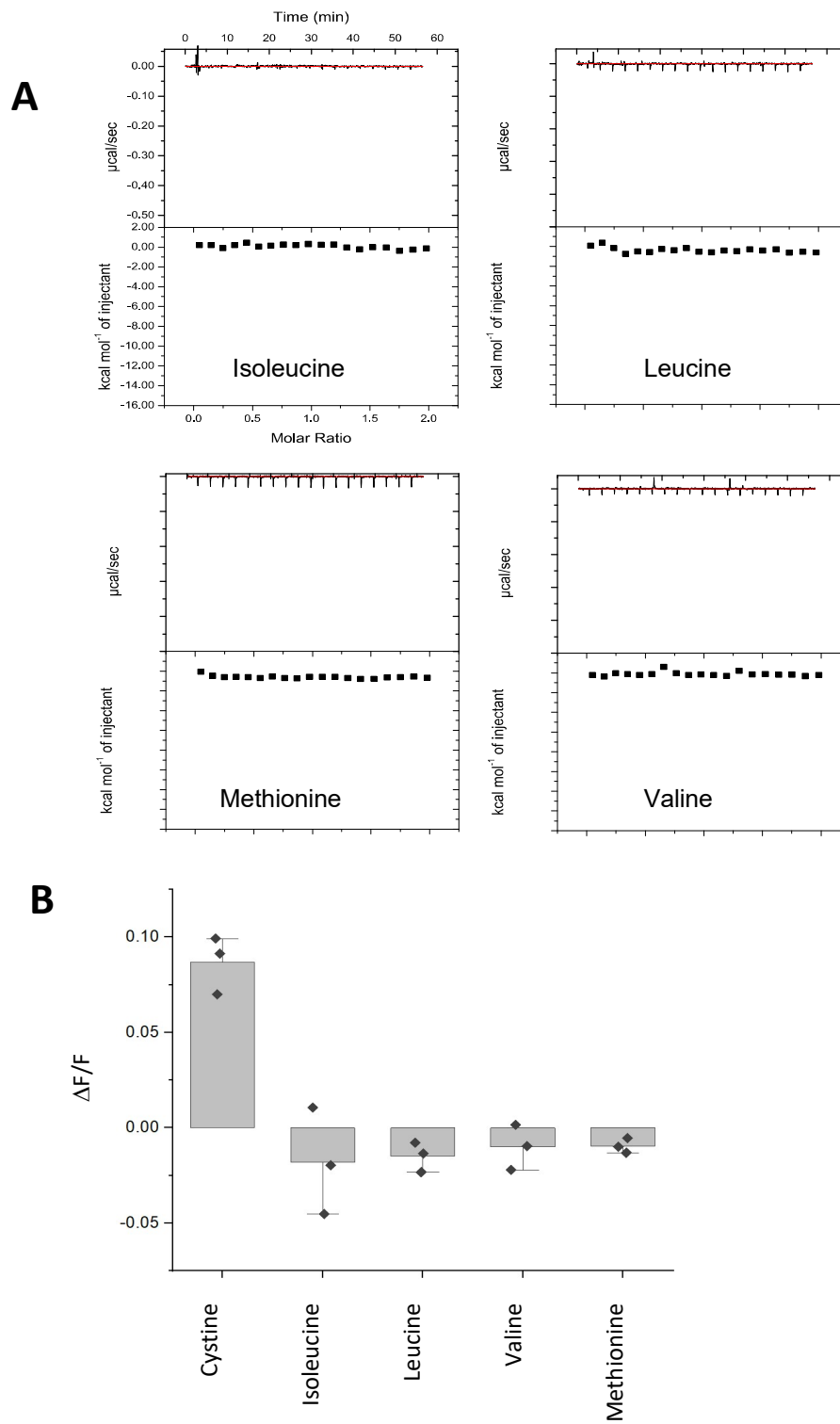

Fig S6

Supplement: FIG S6 [file mbio.00448-22-s0006.pdf]

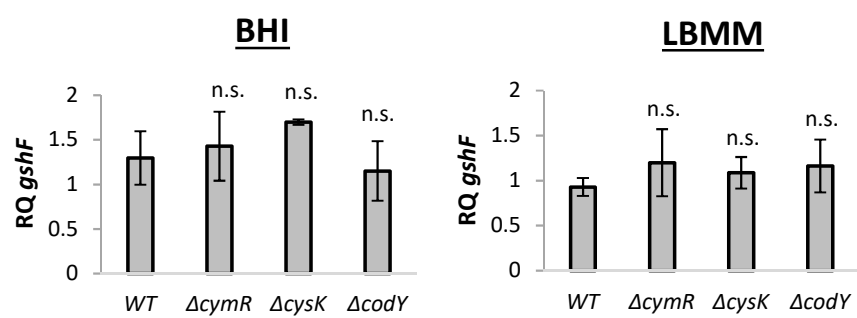

**Fig S7**

Supplement: FIG S7 [file mbio.00448-22-s0007.pdf]

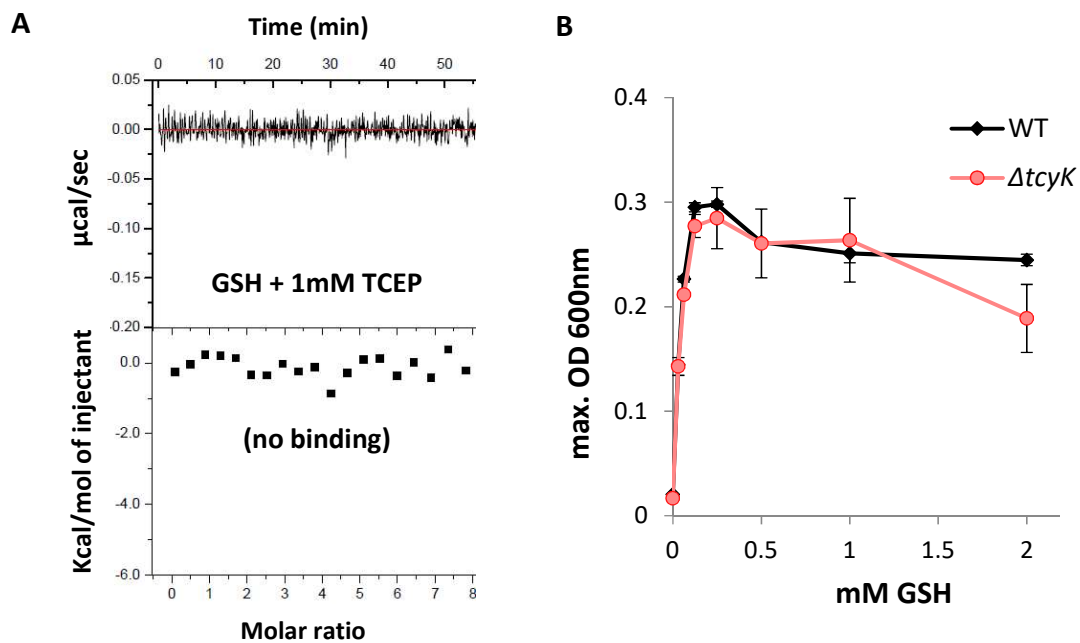

Fig S8

Supplement: FIG S8 [file mbio.00448-22-s0008.pdf]

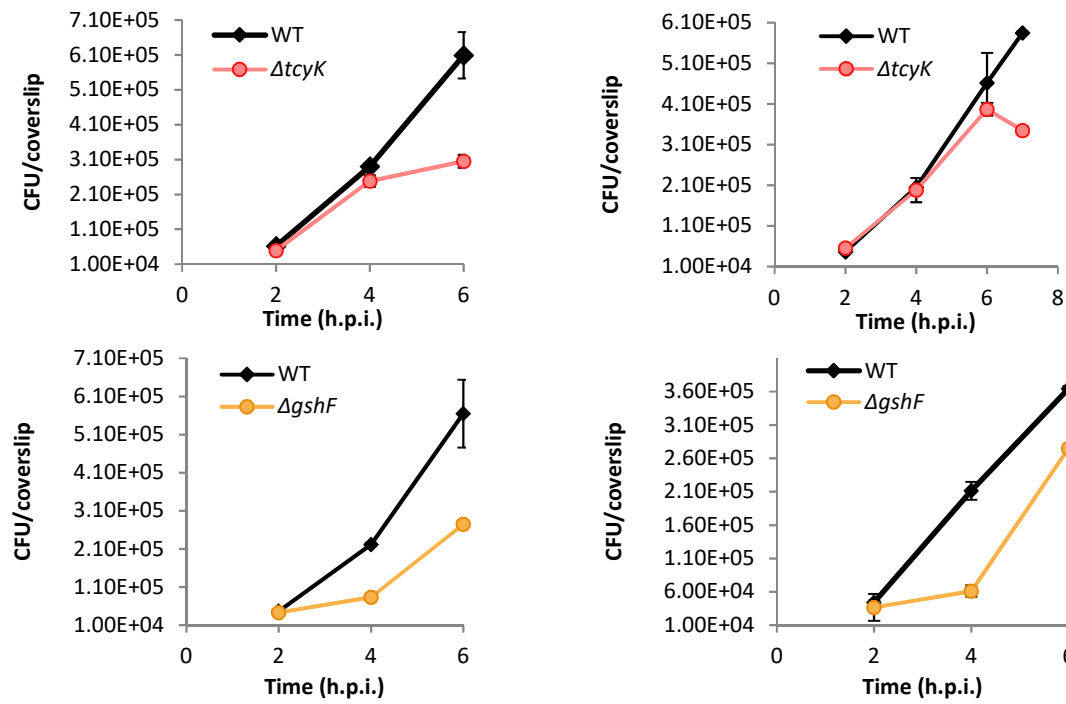

Fig S9

Supplement: FIG S9 [file mbio.00448-22-s0009.pdf]
