# Supplementary material for: Listeria monocytogenes TcyKLMN Cystine/Cysteine Transporter Facilitates Glutathione Synthesis and Virulence Gene Expression
Source: mBio. 2022 Apr 18;13(3):e00448-22. doi: 10.1128/mbio.00448-22 (PMC9239247; doi:10.1128/mbio.00448-22)
Supplement: TABLE S1 [file mbio.00448-22-s0010.docx]

**Table S1. Strains, plasmids and primer used in this study**

| **Strain** | **Description** | **Source** |
| --- | --- | --- |
| *E. coli* XL-1 Blue | Plasmid propagation strain | Stratagene |
| *E. coli* SM-10 | For plasmid conjugation to *L. monocytogenes* | -1 |
| *E. coli* BL21-Gold (DE3) | For over-expression of N'-His-TcyK | Stratagene |
| WT *Lm* | *Listeria monocytogenes* strain *10403S* | D. Portnoy lab |
| *cysK*::*Tn* | *cysK (LMRG_02645)* Himar1 mariner Tn mutant harboring *pPL2-Phly-lux* | -2 |
| *tcyN*::*Tn* | *tcyN (LMRG_01497)* Himar1 mariner Tn mutant harboring *pPL2-Phly-lux* | -2 |
| *ΔtcyK* | *tcyK (LMRG_01494)* deletion mutant | This study |
| *ΔcysK* | *cysK (LMRG_02645)* deletion mutant | This study |
| *ΔcymR* | *cymR (LMRG_01455)* deletion mutant | This study |
| *ΔcodY* | *codY* (*LMRG_00730*) deletion mutant | -3 |
| *ΔgshF* | *gshF (LMRG_01925)* deletion mutant | This study |
| *ΔcysK/ΔgshF* | Double deletion mutant | This study |
| *ΔcymR/ΔgshF* | Double deletion mutant | This study |
| *ΔcysK/ΔtcyK* | Double deletion mutant | This study |
| *ΔcymR/ΔtcyK* | Double deletion mutant | This study |
| WT *Lm pPL2 Phly-lux* | WT *Lm* harboring *pPL2-Phly-lux* | -2 |
| *ΔtcyK pPL2 Phly-lux* | Deletion mutant harboring *pPL2-Phly-lux* | This study |
| *ΔcymR pPL2 Phly-lux* | Deletion mutant harboring *pPL2-Phly-lux* | This study |
| *ΔgshF pPL2 Phly-lux* | Deletion mutant harboring *pPL2-Phly-lux* | This study |
| *ΔcysK/ΔgshF pPL2 Phly-lux* | Double deletion mutant harboring *pPL2-Phly-lux* | This study |
| *ΔcymR/ΔgshF pPL2 Phly-lux* | Double deletion mutant harboring *pPL2-Phly-lux* | This study |
| *ΔcysK/ΔtcyK pPL2 Phly-lux* | Double deletion mutant harboring *pPL2-Phly-lux* | This study |
| *ΔcymR/ΔtcyK pPL2 Phly-lux* | Double deletion mutant harboring *pPL2-Phly-lux* | This study |
| *ΔtcyK pPL2 Ptet-tcyK* | Complementation of deletion mutant | This study |
| *ΔcysK pPL2 Ptet-cysK* | Complementation of deletion mutant | This study |
| WT *Lm pPL2 PplcA-3yfp* | WT *Lm* harboring pPL2-*PplcA-3yfp* | -4 |
| *ΔtcyK pPL2 PplcA-3yfp* | *tcyK* deletion mutant harboring pPL2-*PplcA-3yfp* | This study |
| *ΔprfA pPL2-Phly-lux* | Deletion of *prfA* gene (DPL-4317, Portnoy lab) and *pPL2-Phly-lux* | This study |
| *prfA* pPL2-Phly-lux* | L140F mutation in *prfA* gene (DPL-5450, Portnoy lab) and *pPL2-Phly-lux* | This study |
| *ΔtcyK/prfA* pPL2 Phly-lux* | *tcyK* deletion mutant harboring *prfA** allele and  *pPL2-Phly-lux* | This study |
| *ΔcysK/ΔprfA pPL2 Phly-lux* | *cysK* and *prfA* deletion mutant harboring *pPL2-Phly-lux* | This study |
| **Plasmid** | **Description** | **Source** |
| *pBHE261* (pKS7-oriT) | A conjugative plasmid for delivery to *L. monocytogenes,* used for generating clean deletion strains | Portnoy lab stock, DP-E6324 |
| pPL2 | A chromosomally integrative plasmid of *L. monocytogenes* | -5 |
| pPL2-*Phly-lux* | reporter plasmid for *hly* gene transcription adapted with Km resistance cassette | C. Hill lab and (2) |
| pPL2-*Ptet-tcyK* | *tcyK* under Anhydrotetracycline inducible promoter | This study |
| *pPL2-Ptet-cysK* | *cysK* under Anhydrotetracycline inducible promoter | This study |
| pPL2-*PplcA-3YFP* | Three consecutive YFP proteins under the regulation of the *plcA* promoter | -4 |
| *pET-19b* | An expression vector for N-terminal His-tagged proteins, for expression of TcyK | Novagen |
| **Primer Name** | **Sequence** | **Description** |
| tcyK_A | ATATTCTAGAGTGCATAAAATAAACGCAGAAGCA | For *tcyK* deletion + XbaI site for cloning into pBHE261 |
| tcyK_B | GACATAAAAAAGGGGGAAAAAAGATGTATTAAAAGAGAGGGAGTGCTATTAAAATG | For *tcyK* deletion |
| tcyK_C | CATTTTAATAGCACTCCCTCTCTTTTAATACATCTTTTTTCCCCCTTTTTTATGTC | For *tcyK* deletion |
| tcyK_D | ATATCTGCAGATCCACCAATAGGGGAATGC | For *tcyK* deletion + PstI site for cloning into pBHE261 |
| cysK_A | ATATCTGCAGGCTAAACTAGCGGGCG | For *cysK* deletion + PstI site for cloning into pBHE261 |
| cysK_B | GCTCTCTGATTATTACATTATTAAAGCACTCCTATCTATTTTAGGT | For *cysK* deletion |
| cysK_C | GTGCTTTAATAATGTAATAATCAGAGAGCCTAGATTTTCTAGGT | For *cysK* deletion |
| cysK_D | TATAGGATCCTCTCGGTTTTTCTTTTCATTTTTTGC | For *cysK* deletion + BamHI site for cloning into pBHE261 |
| cymR_A | ATAACAATTTCACACAGGAAACAGCTATGACATGATTACGAATTCAGCAATCGCACTTGC | For *cymR* deletion, for Gibson assembly cloning into pBHE261 |
| cymR_B | TAAATAGGCTTTTTTCATTACATTTTTATTTAACCTCCAGTAATTTCA | For *cymR* deletion |
| cymR_C | CTGGAGGTTAAATAAAAATGTAATGAAAAAAGCCTATTTACGAATAGG | For *cymR* deletion |
| cymR_D | TTTTCCCAGTCACGACGTTGTAAAACGACGGCCAGTGCCAAGCTTGCGTCGAACCATACC | For *cymR* deletion, for Gibson assembly cloning into pBHE261 |
| gshF_A | ATATCTGCAGAATCGAACATTGAGTGAAAAAG | For *gshF* deletion + PstI site for cloning into pBHE261 |
| gshF_B | TGTAGCTGGGTTTTTTTACATGTTTTCACCTCAGGGG | For *gshF* deletion |
| gshF_C | TGAGGTGAAAACATGTAAAAAAACCCAGCTACAGTTTAGT | For *gshF* deletion |
| gshF_D | ATATGGATCCCGAGAAGGCACGGTT | For *gshF* deletion + BamHI site for cloning into pBHE261 |
| IndTetR_tcyK_F | ATATGTCGACATGAAAAAGAAATATGGGATTTTGGCG | For *tcyK* complementation + SalI site for cloning into pPL2 IndtetR |
| IndTetR_tcyK_R | ATATCTGCAGTTAATATTGTTCTTTTGAATAATCTGCGCC | For *tcyK* complementation + PstI site for cloning into pPL2 IndtetR |
| IndTetR_cysK_F | ATATGTCGACATGACAATTGCAAATTCAATCACTGA | For *cysK* complementation + SalI site for cloning into pPL2 IndtetR |
| IndTetR_cysK_R | ATATCTGCAGTTAATCTTCAAAATTATAAAGCGGCGTG | For *cysK* complementation + PstI site for cloning into pPL2 IndtetR |
| **Primer Name** | **Sequence** |  |
| rpoD_F | GGGGCTAATGAAAGCCGTTG |  |
| rpoD_R | CGCTTGACGAATCCACCACG |  |
| tcyK_F | CTGTCTAATACCGTCAATTGTAATG |  |
| tcyK_R | GGATTTTTCTAACTTACTCGTTAGCC |  |
| tcyN_F | CACCGACAAGTTCAGGATCA |  |
| tcyN_R | AGTGCCTACCCTTCCCAACT |  |
| ytlI_F | GCGAATAAAGCAATTGGAACA |  |
| ytlI_R | CCCGCCCTTTCGTAAAATAA |  |
| gshF_F | ACAGGTGGCGATAGCATTGA |  |
| gshF_R | CAATTATGTCTACGCCGCAAA |  |
| cysK_F | TAGTGGCGGTTCTCCATCTC |  |
| cysK_R | TTCTAGGGCATCTTCGCTTG |  |
